# Supplementary material for: Long noncoding RNA AGPG regulates PFKFB3-mediated tumor glycolytic reprogramming
Source: Nat Commun. 2020 Mar 20;11:1507. doi: 10.1038/s41467-020-15112-3 (PMC7083971; doi:10.1038/s41467-020-15112-3)
Supplement: Supplementary file 1 — Supplementary Information [file 41467_2020_15112_MOESM1_ESM.pdf]

Supplementary Information

Long Noncoding RNA *AGPG* Regulates PFKFB3-Mediated Tumor Glycolytic Reprogramming

Liu et al.

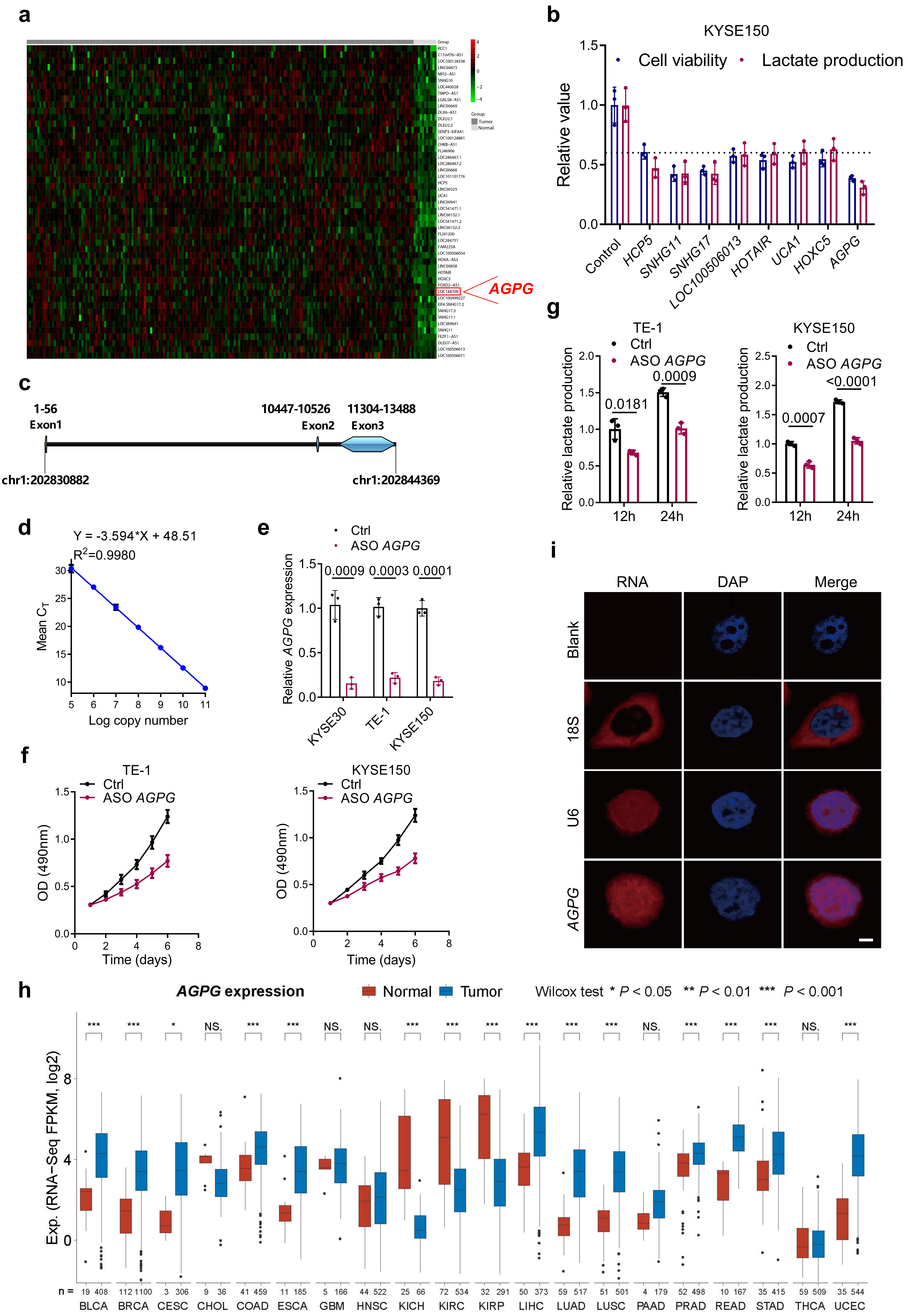

a
AGPG
Group
Tumor
Normal
b
KYSE150
Cell viability
Lactate production
Relative value
Control
HCP5
SNHG11
SNHG17
LOC100506013
HOTAIR
UCA1
HOXC5
AGPG
c
1-56
Exon1
10447-10526
Exon2
11304-13488
Exon3
chr1:202830882
chr1:202844369
d
Y = -3.594*X + 48.51
R^2=0.9980
Mean C_T
Log copy number
e
Ctrl
ASO AGPG
Relative AGPG expression
0.0009
0.0003
0.0001
KYSE30
TE-1
KYSE150
f
TE-1
KYSE150
Ctrl
ASO AGPG
OD (490nm)
Time (days)
g
TE-1
KYSE150
Ctrl
ASO AGPG
Relative lactate production
0.0181
0.0009
0.0007
<0.0001
12h
24h
i
RNA
DAP
Merge
Blank
18S
U6
AGPG
h
AGPG expression
Normal
Tumor
Wilcox test * P < 0.05 ** P < 0.01 *** P < 0.001
Exp. (RNA−Seq FPKM, log2)
n = 19 408 112 1100 3 306 9 36 41 459 11 185 5 166 44 522 25 66 72 534 32 291 50 373 59 517 51 501 4 179 52 498 10 167 35 415 59 509 35 544
BLCA BRCA CESC CHOL COAD ESCA GBM HNSC KICH KIRC KIRP LIHC LUAD LUSC PAAD PRAD READ STAD THCA UCEC

Supplementary Figure 1 Identification of *AGPG* as a metabolism-related lncRNA. (**a**) Top 50 lncRNAs that are highly expressed in ESCC according to the TCGA. (**b**) Eight lncRNAs regulated both cell proliferation and lactate production in KYSE150 cells. (**c**) *AGPG* is located on chromosome 1q32.1 and has 3 exons (1-56, 10447-10526, and 11304-13488). (**d**) Determination of *AGPG* copy number. The standard curve for calculating *AGPG* copy number is shown. *In vitro*-transcribed *AGPG* was subjected to reverse transcription followed by qPCR. The standard curve shows that CT values decreased linearly with increasing *AGPG* copy number, suggesting sensitive detection over a wide range of template amounts (from 10^5 copies to 10^{11} copies). Then, the data were converted into copy number per cell based on the known input cell number. (**e**) *AGPG* expression in ESCC cells transduced with ASO AGPG or Ctrl. (**f**) TE-1 and KYSE150 cell proliferation was assessed by MTS assays (OD 490 nm). (**g**) Lactate production was assessed in TE-1 and KYSE150 cells after culture for 12 h and 24 h. (**h**) Analysis of AGPG expression in multiple human cancers from the TCGA. Data are presented as a box-and-whiskers graph: the bottom and top of the box are the lower and upper quartiles, the band near the middle of the box is the median, the ends of the whiskers represent the minimum and maximum of the data. The *P* value was determined by a two-sided Wilcox test, sample sizes have been indicated in the figure, * $P < 0.05$ ** $P < 0.01$ *** $P < 0.001$. (**i**) The subcellular localization of *AGPG* detected by RNA FISH in KYSE150 cells. Scale bar: 5 μm. Data in **b,d-g** are representative of three independent experiments and presented as mean ± S.D., n=3 biologically independent samples, the *P* value was determined by a two-tailed unpaired Student’s *t* test.

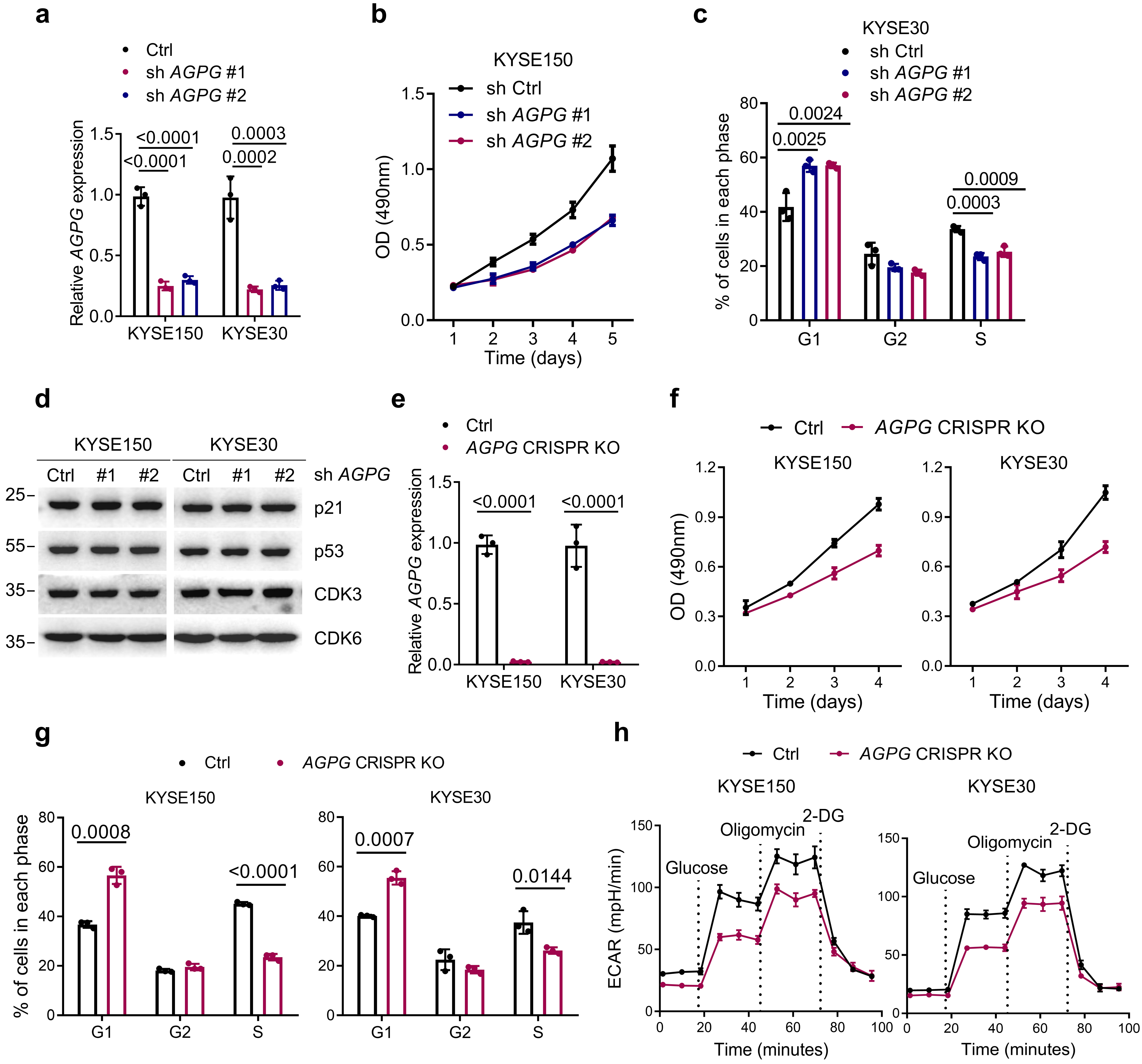

Supplementary Figure 2 *AGPG* is required for cell proliferation and metabolism remodeling. (**a**) *AGPG* expression in ESCC cells transduced with sh*AGPG* or shCtrl. (**b**) KYSE150 cell proliferation was assessed by MTS assays (OD 490 nm). (**c**) Statistical analysis of the percentage (%) of KYSE30 cells in each cell cycle phase. (**d**) p21, p53, CDK3 and CDK6 expression levels were detected by western blotting in ESCC cells transduced with sh*AGPG* or shCtrl. (**e**) *AGPG* CRISPR KO cells were generated using the CRISPR/Cas9 genome editing system. KO, knockout. (**f**) ESCC cell proliferation was assessed by MTS assays (OD 490 nm). *AGPG* CRISPR KO strikingly inhibited cell proliferation. (**g**) Cell cycle analysis showed that *AGPG* CRISPR KO blocked the G1/S cell cycle transition. (**h**) The ECAR was measured in ESCC cells using an XF Extracellular Flux Analyzer. *AGPG* CRISPR KO

significantly impaired glycolysis. Data in **a-c,e-h** are representative of three independent experiments and presented as mean ± S.D., n=3 biologically independent samples, the *P* value in **a-c** was determined by one-way ANOVA with Dunnett's multiple comparisons test, no adjustments were made for multiple comparisons. The *P* value in **e-g** was determined by a two-tailed unpaired Student's *t* test.

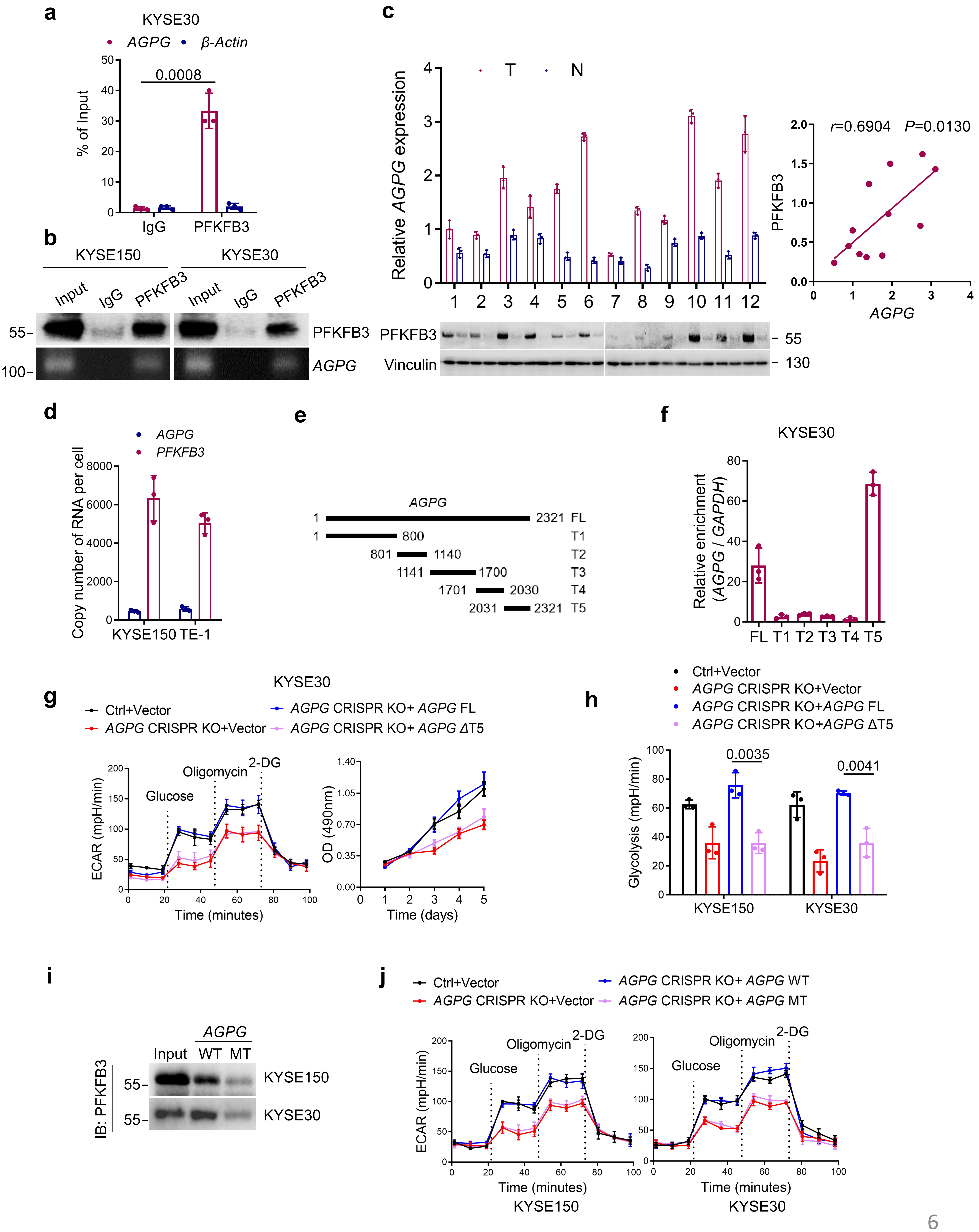
a
KYSE30
AGPG β-Actin
0.0008
% of Input
IgG PFKFB3
b
KYSE150 KYSE30
Input IgG PFKFB3 Input IgG PFKFB3
55–
100–
PFKFB3
AGPG
c
T N
Relative AGPG expression
1 2 3 4 5 6 7 8 9 10 11 12
PFKFB3
Vinculin
– 55
– 130
r=0.6904 P=0.0130
PFKFB3
AGPG
d
AGPG
PFKFB3
Copy number of RNA per cell
KYSE150 TE-1
e
AGPG
1 2321 FL
1 800 T1
801 1140 T2
1141 1700 T3
1701 2030 T4
2031 2321 T5
f
KYSE30
Relative enrichment (AGPG / GAPDH)
FL T1 T2 T3 T4 T5
g
KYSE30
Ctrl+Vector
AGPG CRISPR KO+Vector
AGPG CRISPR KO+ AGPG FL
AGPG CRISPR KO+ AGPG ΔT5
Glucose
Oligomycin
2-DG
ECAR (mpH/min)
Time (minutes)
OD (490nm)
Time (days)
h
Ctrl+Vector
AGPG CRISPR KO+Vector
AGPG CRISPR KO+AGPG FL
AGPG CRISPR KO+AGPG ΔT5
0.0035
0.0041
Glycolysis (mpH/min)
KYSE150 KYSE30
i
AGPG
Input WT MT
IB: PFKFB3
55–
55–
KYSE150
KYSE30
j
Ctrl+Vector
AGPG CRISPR KO+Vector
AGPG CRISPR KO+ AGPG WT
AGPG CRISPR KO+ AGPG MT
Glucose
Oligomycin
2-DG
ECAR (mpH/min)
Time (minutes)
KYSE150
KYSE30

Supplementary Figure 3 *AGPG* directly associates with PFKFB3. (**a,b**) RIP assays indicated that *AGPG* precipitated with PFKFB3 in whole-cell lysates. *AGPG* RNA levels were measured by qPCR (a) and electrophoresis detection (b). (**c**) qPCR detection of *AGPG* expression and western blotting detection of PFKFB3 expression in a cohort of ESCC patients. PFKFB3 expression was positively correlated with *AGPG* expression in ESCC patients (Pearson's correlation analysis, n = 12). (**d**) Absolute quantitation of *AGPG* and *PFKFB3* copy number by digital droplet PCR. (**e**) Schematic diagram of the *AGPG* truncation mutants. (**f**) CLIP-qPCR showed that the T5 fragment of *AGPG* was the region responsible for PFKFB3 binding. (**g**) Overexpression of *AGPG* FL, but not of *AGPG* ΔT5, was sufficient to reverse the decreased ECAR and proliferation caused by *AGPG* CRISPR KO in KYSE30 cells. (**h**) Overexpression of *AGPG* FL, but not of *AGPG* ΔT5, was sufficient to rescue the reduced glycolysis caused by *AGPG* CRISPR KO. (**i**) RNA pull-down assays showed that the binding of a mutant lacking the CCAGCCA motif (*AGPG* MT) and PFKFB3 was significantly reduced. (**j**) Overexpression of *AGPG* MT could not rescue the decreased glycolysis caused by *AGPG* KO. Data in **a,c,d,f-h,j** are representative of three independent experiments and presented as mean ± S.D., n=3 biologically independent samples, the *P* value was determined by a two-tailed unpaired Student's *t* test.

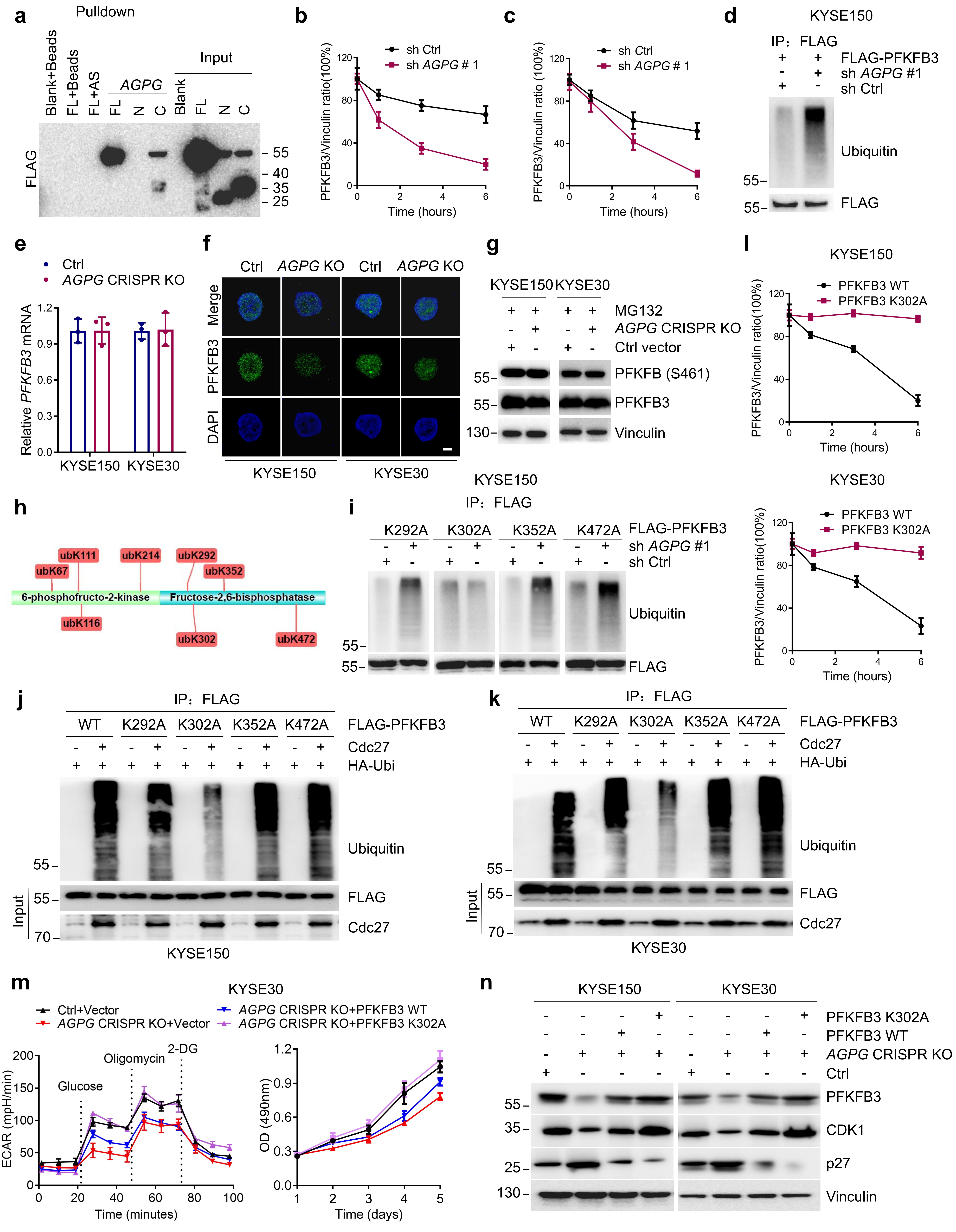
a
Pulldown
Blank+Beads
FL+Beads
FL+AS
AGPG
FL
N
C
Input
Blank
FL
N
C
FLAG
55
40
35
25
b
PFKFB3/Vinculin ratio(100%)
sh Ctrl
sh AGPG # 1
Time (hours)
c
PFKFB3/Vinculin ratio (100%)
sh Ctrl
sh AGPG # 1
Time (hours)
d
KYSE150
IP：FLAG
FLAG-PFKFB3
sh AGPG #1
sh Ctrl
Ubiquitin
FLAG
55
e
Ctrl
AGPG CRISPR KO
Relative PFKFB3 mRNA
KYSE150
KYSE30
f
Ctrl
AGPG KO
Ctrl
AGPG KO
Merge
PFKFB3
DAPI
KYSE150
KYSE30
g
KYSE150
KYSE30
MG132
AGPG CRISPR KO
Ctrl vector
PFKFB (S461)
PFKFB3
Vinculin
55
55
130
l
KYSE150
PFKFB3 WT
PFKFB3 K302A
PFKFB3/Vinculin ratio(100%)
Time (hours)
KYSE30
PFKFB3 WT
PFKFB3 K302A
PFKFB3/Vinculin ratio(100%)
Time (hours)
h
ubK111
ubK214
ubK292
ubK67
ubK352
6-phosphofructo-2-kinase
Fructose-2,6-bisphosphatase
ubK116
ubK302
ubK472
i
KYSE150
IP：FLAG
K292A
K302A
K352A
K472A
FLAG-PFKFB3
sh AGPG #1
sh Ctrl
Ubiquitin
FLAG
55
j
IP：FLAG
WT
K292A
K302A
K352A
K472A
FLAG-PFKFB3
Cdc27
HA-Ubi
Ubiquitin
Input
FLAG
Cdc27
55
70
KYSE150
k
IP：FLAG
WT
K292A
K302A
K352A
K472A
FLAG-PFKFB3
Cdc27
HA-Ubi
Ubiquitin
Input
FLAG
Cdc27
55
70
KYSE30
m
KYSE30
Ctrl+Vector
AGPG CRISPR KO+Vector
AGPG CRISPR KO+PFKFB3 WT
AGPG CRISPR KO+PFKFB3 K302A
ECAR (mpH/min)
Glucose
Oligomycin
2-DG
Time (minutes)
OD (490nm)
Time (days)
n
KYSE150
KYSE30
PFKFB3 K302A
PFKFB3 WT
AGPG CRISPR KO
Ctrl
PFKFB3
CDK1
p27
Vinculin
55
35
25
130

Supplementary Figure 4 *AGPG* affects PFKFB3 stability by preventing its ubiquitination. (**a**) *In vitro*-synthesized *AGPG* was incubated with protein lysates from KYSE150 cells transfected with vectors expressing FLAG-tagged FL or truncation mutants of PFKFB3. RNA pull-down assays were performed using streptavidin beads, and the products were subjected to western blotting. (**b,c**) Western blotting detection of PFKFB3 levels in ESCC cells transfected with shCtrl or sh*AGPG* and treated with CHX (100 μg per ml) for the indicated times. Quantification of PFKFB3 protein levels. (**d**) IP assays showed that *AGPG* knockdown increased PFKFB3 ubiquitination in KYSE150 cells. (**e**) *AGPG* CRISPR KO did not affect PFKFB3 mRNA levels. (**f**) *AGPG* CRISPR KO did not affect PFKFB3 subcellular localization. Scale bar: 5 μm. (**g**) Cells were treated with MG-132 (10 μM, 12 h) before being subjected to western blotting. *AGPG* CRISPR KO did not affect PFKFB3 S461 phosphorylation. (**h**) Schematic diagram of the ubiquitination sites on PFKFB3. Data on the post-translational modification of PFKFB3 were obtained from the dbPAF and CPLM databases. (**i**) IP assays showed that *AGPG* knockdown did not increase PFKFB3 K302A ubiquitination. (**j**) KYSE150 cells were transfected with the indicated plasmids. Cells were then immunoprecipitated with a Flag antibody and subjected to western blotting with a ubiquitin antibody. (**k**) KYSE30 cells were transfected with the indicated plasmids. Cells were then immunoprecipitated with a Flag antibody and subjected to western blotting with a ubiquitin antibody. (**l**) Cells with stable expression of sh*AGPG* were infected with FLAG-tagged PFKFB3 WT or K302A and treated with CHX (100 μg per ml) for the indicated time. PFKFB3 protein levels were quantified. (**m**) PFKFB3 K302A overexpression in KYSE30 cells significantly reversed the decreased ECAR and proliferation caused by *AGPG* CRISPR KO, while PFKFB3 WT only partially rescued these effects. (**n**) Western blotting showed that PFKFB3 K302A overexpression rescued the *AGPG* CRISPR KO-mediated decrease in CDK1 and increase in p27, while PFKFB3 WT only partially rescued these effects. Data in **b,c,e,l,m** are representative of three independent experiments and presented as mean ± S.D., n=3 biologically independent samples.

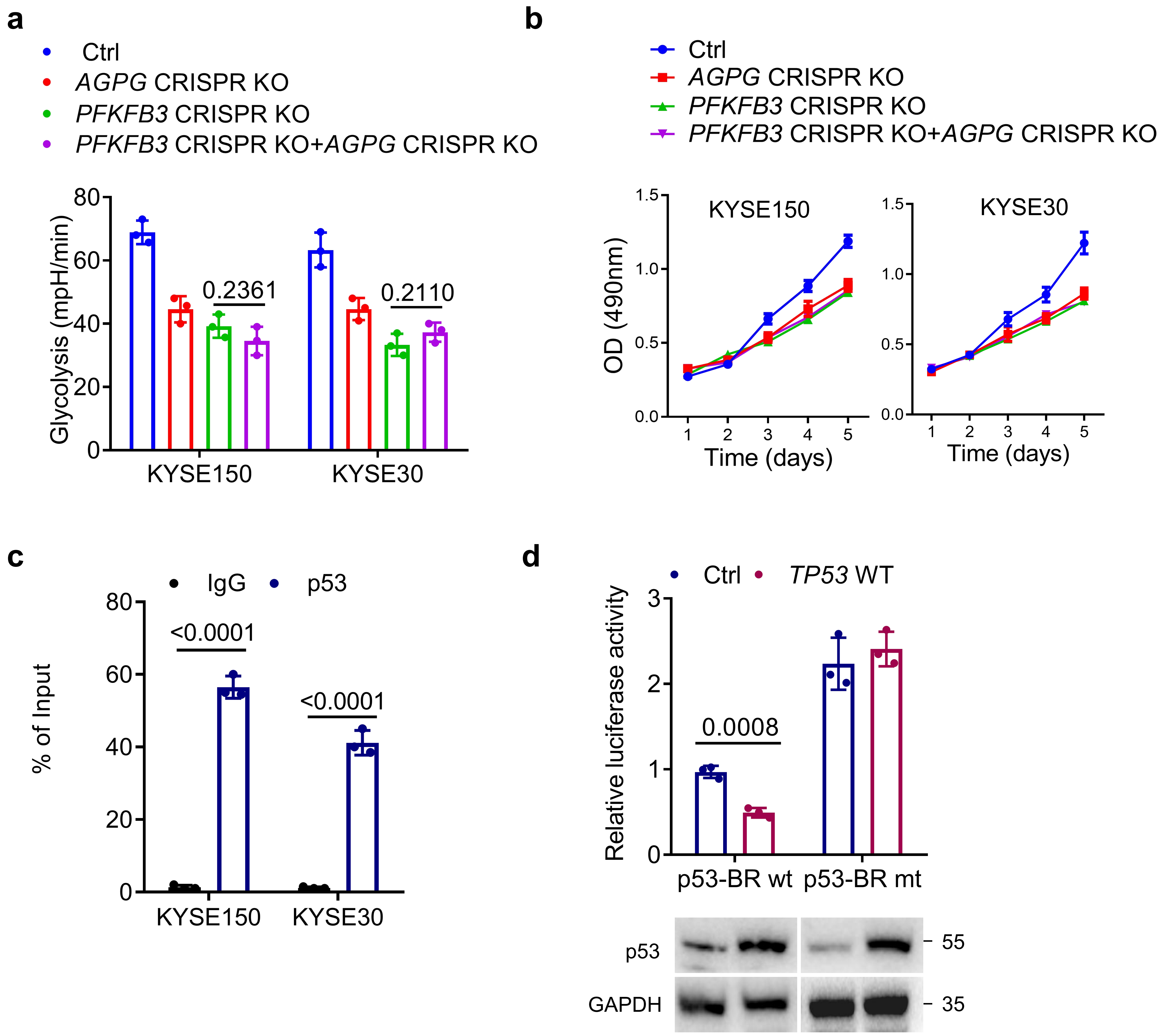

Supplementary Figure 5 *AGPG* is transcriptionally regulated by p53. (a,b) After *PFKFB3* KO in ESCC cells, *AGPG* CRISPR KO had mild effects on aerobic glycolysis (a) and cell proliferation (b). (**c**) ChIP assays showed that p53 bound to the *AGPG* promoter. (**d**) WT p53 overexpression diminished the transcription of *AGPG* in HCT-116 cells. Data are representative of three independent experiments and presented as mean±S.D., n=3 biologically independent samples, the *P* value was determined by a two-tailed unpaired Student's *t* test.

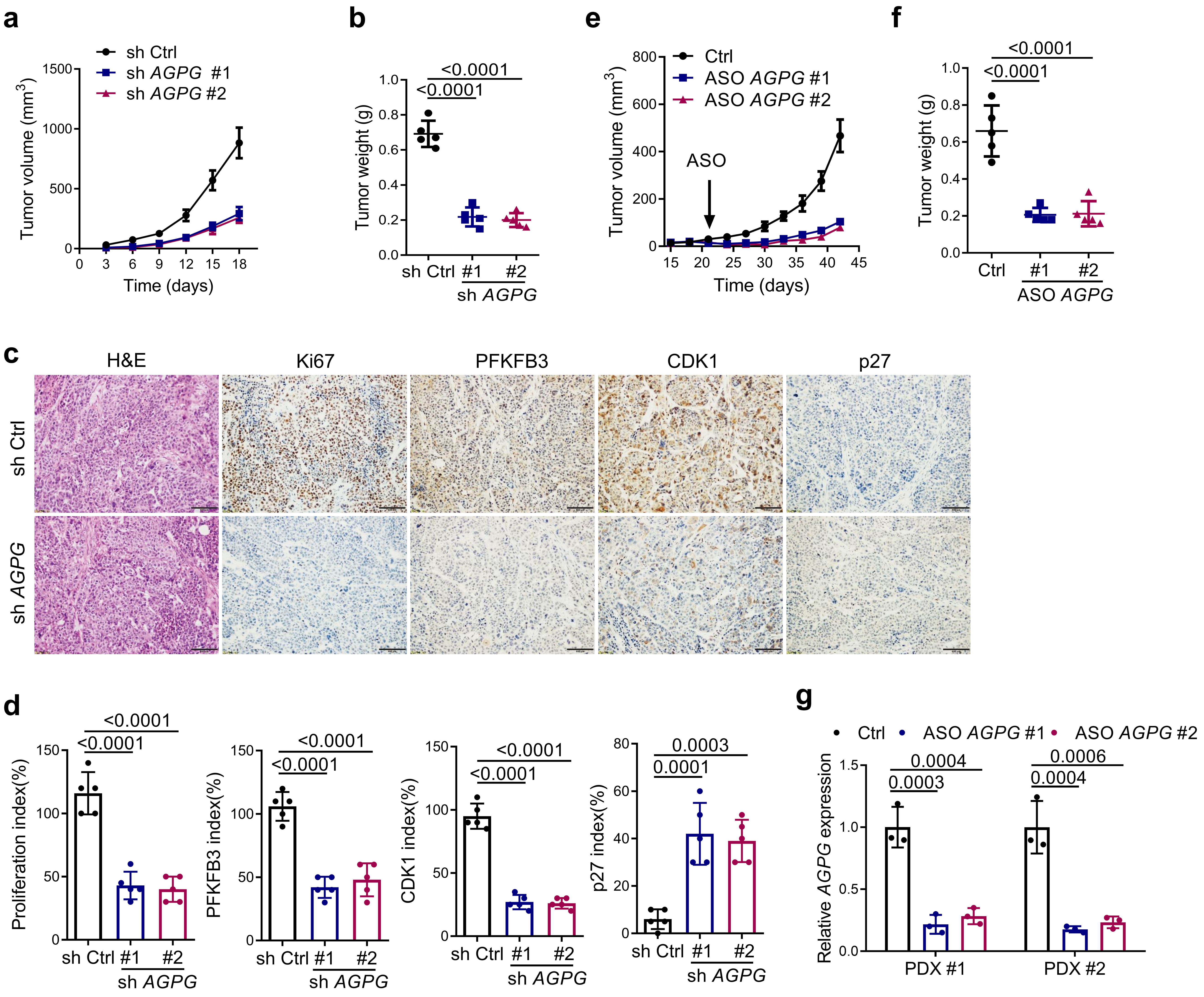

Supplementary Figure 6 Effects of *AGPG* on tumor growth *in vivo*. (**a, b**) Statistical analysis of KYSE30 tumor volume and weight in nude mice. (**c**) Representative IHC images of randomly selected KYSE30 cell-based tumors from each group are shown. Scale bar, 100 μm. (**d**) Quantification of IHC staining of KYSE30 cell-based tumors. (**e, f**) Statistical analysis of tumor volume and weight in the PDX #2 model. (**g**) qPCR analysis of *AGPG* expression in PDX tumor tissues treated with scrambled or *in vivo*-optimized *AGPG* inhibitor. Data are representative of three independent experiments and presented as mean ± S.D., n=5 mice per group, the *P* value was determined by one-way ANOVA with Dunnett's multiple comparisons test, no adjustments were made for multiple comparisons.

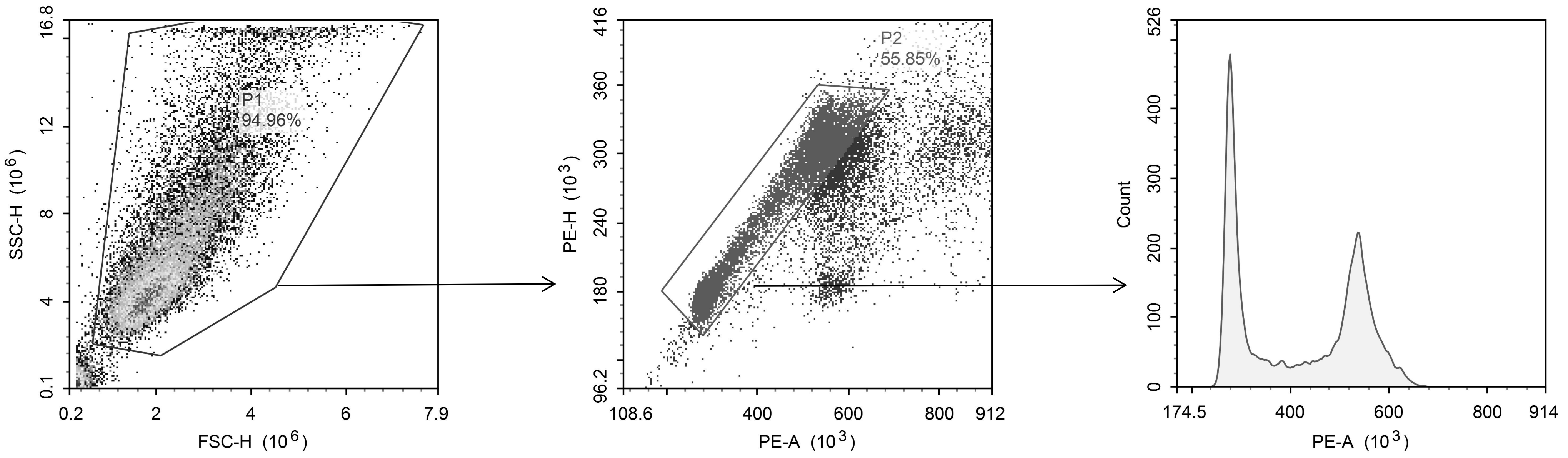

Supplementary Figure 7 The gating strategy for cell cycle analysis. The gating strategy was used for cell cycle analysis in this study.

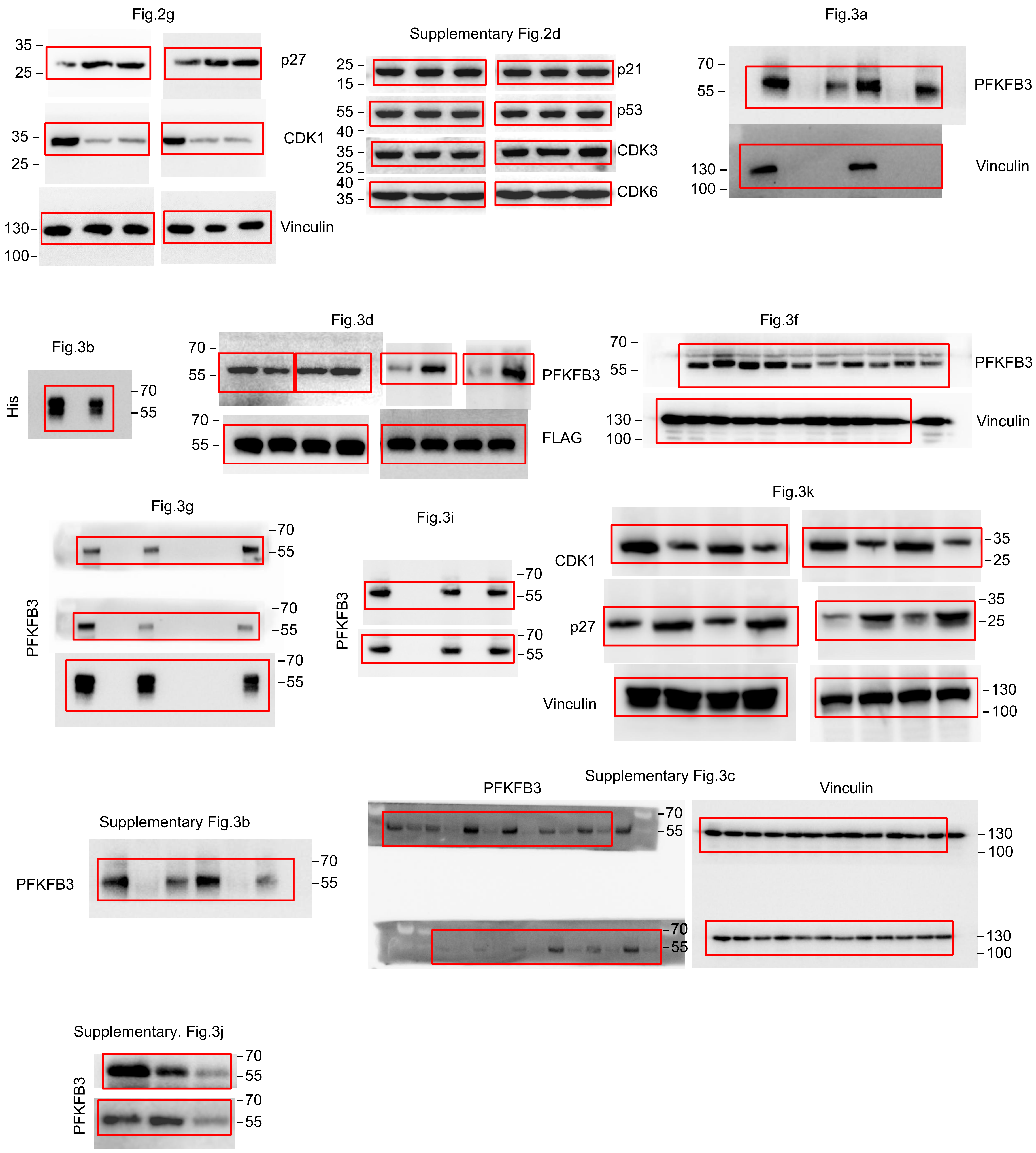
Fig.2g
35 –
25 –
p27
35 –
25 –
CDK1
130–
100–
Vinculin
Supplementary Fig.2d
25 –
15 –
p21
55 –
40 –
p53
35 –
25 –
CDK3
40 –
35 –
CDK6
Fig.3a
70 –
55 –
PFKFB3
130 –
100 –
Vinculin
Fig.3b
His
–70
–55
Fig.3d
70 –
55 –
PFKFB3
70 –
55 –
FLAG
Fig.3f
70 –
55 –
PFKFB3
130 –
100 –
Vinculin
Fig.3g
PFKFB3
–70
–55
–70
–55
–70
–55
Fig.3i
PFKFB3
–70
–55
–70
–55
Fig.3k
CDK1
–35
–25
p27
–35
–25
Vinculin
–130
–100
Supplementary Fig.3b
PFKFB3
–70
–55
Supplementary Fig.3c
PFKFB3
–70
–55
–70
–55
Vinculin
–130
–100
–130
–100
Supplementary. Fig.3j
PFKFB3
–70
–55
–70
–55

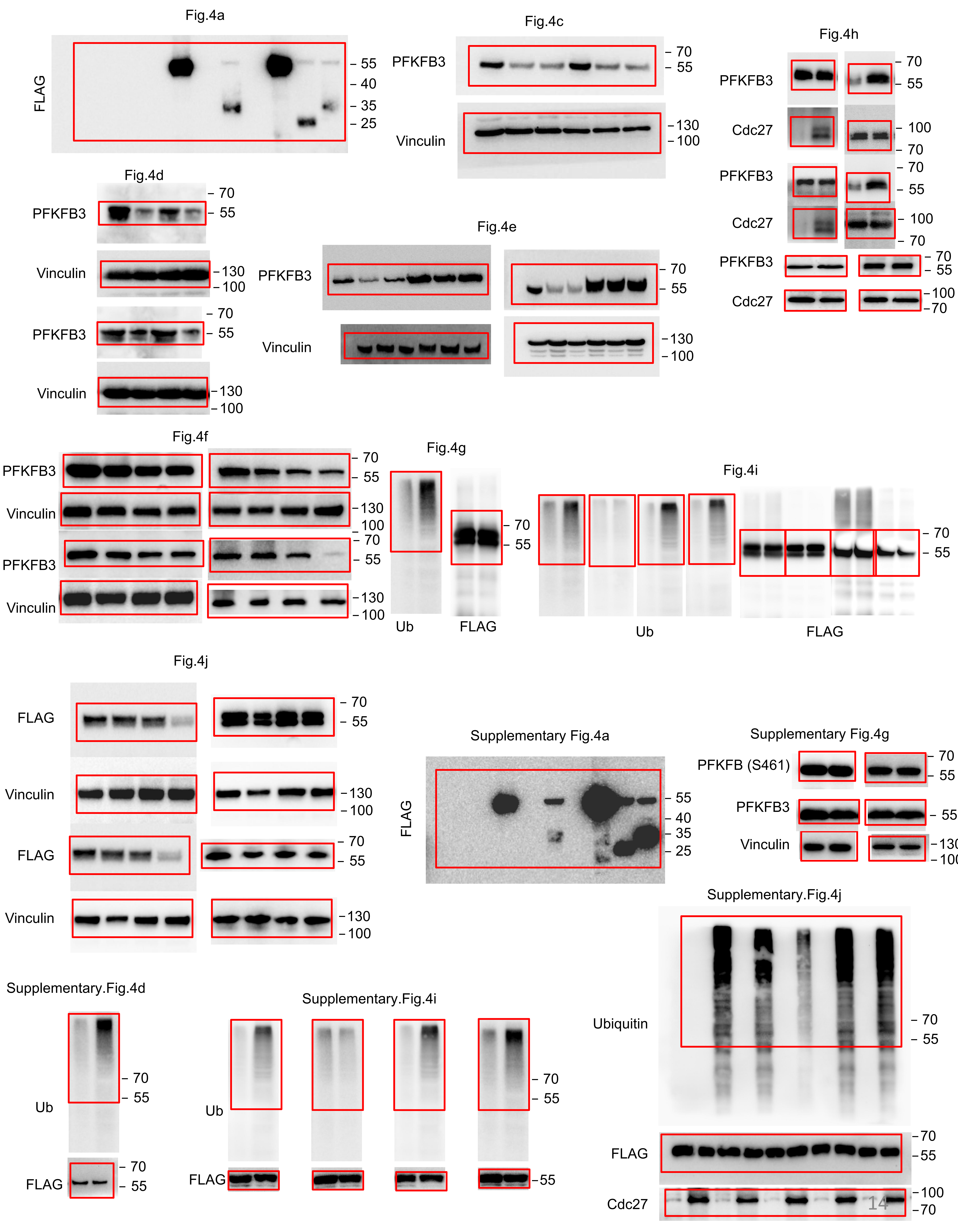

Fig.4a
FLAG
55
40
35
25
Fig.4c
PFKFB3
Vinculin
70
55
130
100
Fig.4h
PFKFB3
Cdc27
PFKFB3
Cdc27
PFKFB3
Cdc27
70
55
100
70
Fig.4d
PFKFB3
Vinculin
PFKFB3
Vinculin
70
55
130
100
Fig.4e
PFKFB3
Vinculin
70
55
130
100
Fig.4f
PFKFB3
Vinculin
PFKFB3
Vinculin
70
55
130
100
Fig.4g
Ub
FLAG
70
55
Fig.4i
Ub
FLAG
70
55
Fig.4j
FLAG
Vinculin
FLAG
Vinculin
70
55
130
100
Supplementary Fig.4a
FLAG
55
40
35
25
Supplementary Fig.4g
PFKFB (S461)
PFKFB3
Vinculin
70
55
130
100
Supplementary.Fig.4j
Ubiquitin
FLAG
Cdc27
70
55
100
Supplementary.Fig.4d
Ub
FLAG
70
55
Supplementary.Fig.4i
Ub
FLAG
70
55

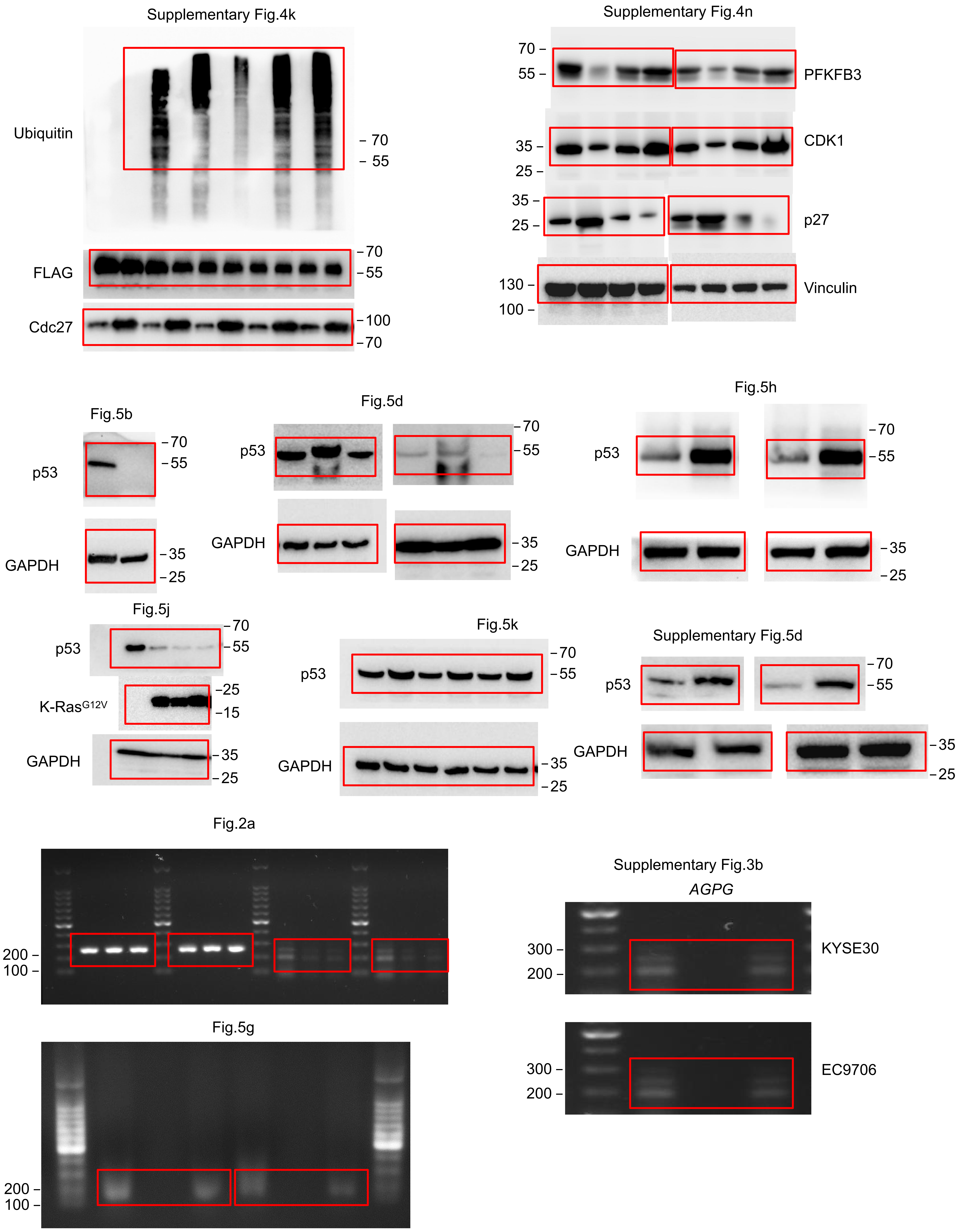

Supplementary Figure 8 Full scan figure for the blots. The red sections indicate blot results shown in the indicated figures.

Supplementary Table 1. Correlation between *AGPG* expression and clinicopathological features in 122 ESCC patients

Characteristics	Low expression n = 61	High expression n = 61	P value
Age			
<59	30 (49.2%)	31(50.8%)	0.856
≥59	31 (50.8%)	30(49.2%)	
Gender			
Male	50 (82.0%)	44 (72.1%)	0.196
Female	11 (18.0%)	17 (27.9%)	
Differentiation status			
Well/Moderate	49 (80.3%)	51 (83.6%)	0.638
Poor and others	12 (19.7%)	10 (16.4%)	
Tumor depth			
m/sm/mp	14 (23.0%)	13 (21.3%)	0.827
ss/se/si	47 (77.0%)	48 (78.7%)	
Lymph node invasion			
Absent	32 (52.5%)	24 (39.3%)	0.146
Present	29(47.5%)	37 (60.7%)	
Vascular invasion			
Absent	58 (95.1%)	60(98.4%)	0.611
Present	3 (4.9%)	1(1.6%)	
Distant metastasis			
Absent	53(86.9%)	52(85.2%)	0.794
Present	8(13.1%)	9(14.8%)	
Clinical stage			
I, II	36(59.0%)	27(44.3%)	0.103
III, IV	25(41.0%)	34 (55.7%)	

Supplementary Table 1. Correlation between *AGPG* expression and clinicopathological features in 122 ESCC patients. The *P* value was determined by a Chi-square test. All the statistical tests were two-sided. Abbreviations: m: tumor invasion of mucosa; sm: submocosa; mp: muscularis propria; ss: subserosa; se: serosa penetration; si: invasion to adjacent structures.

Supplementary Table 2. Effect of factors on overall survival in ESCC patients in the univariate and multivariate cox regression model

Factors	Univariate		Multivariate	
	HR (95% CI)	P value	HR (95% CI)	P value
Age	1.349 (0.818-2.225)	0.241	1.943（1.156-3.266）	0.012
Gender	1.264 (0.716-2.233)	0.420	-	-
Differentiation	1.208 (0.643-2.269)	0.558	-	-
Clinical Stage	3.367 (1.966-5.765)	0.000	3.710（2.132-6.456）	0.000
Tumor depth	2.872 (1.306-6.315)	0.009	-	-
Lymph node invasion	3.010 (1.733-5.229)	0.000	-	-
Vascular invasion	2.425 (0.757-7.767)	0.136	-	-
Distant metastasis	1.376 (0.698-2.711)	0.356	-	-
AGPG	1.885 (1.134-3.134)	0.015	1.816 (1.084-3.042)	0.023

Supplementary Table 2. Effect of factors on overall survival in ESCC patients in the univariate and multivariate cox regression model. The *P* value was determined by the univariate and multivariate cox regression analysis. All the statistical tests were two-sided. Abbreviations: m: tumor invasion of mucosa; sm: submocosa; mp: muscularis propria; ss: subserosa; se: serosa penetration; si: invasion to adjacent structures.

Supplementary Table 3. The RNA motifs recognized by PFKFB3

Rank	Piranha	Cims
1	CCAGSYA	AAAAAAA
2	CTTGAAC	CCAKCCA
3	GTCAACA	TAGYTG
4	TATAGTY	TCYVTCT
5	AGGCTGA	GTCTC

Supplementary Table 4. Correlation between PFKFB3 expression and clinicopathological features in 104 ESCC patients

Characteristics	Low expression n=52	High expression n=52	P value
Age			
<58	29 (55.8%)	30(57.7%)	0.843
≥58	23 (44.2%)	22(42.3%)	
Gender			
Female	14 (26.9%)	17(32.7%)	0.520
Male	38 (73.1%)	35 (67.3%)	
Differentiation status			
Well/Moderate	44 (84.6%)	44 (84.6%)	0.969
Poor and others	8(15.4%)	8(15.4%)	
Tumor depth			
m/sm/mp	18 (34.6%)	12 (23.1%)	0.194
ss/se/si	34 (65.4%)	40 (76.9%)	
Lymph node invasion			
Absent	29 (55.8%)	31 (59.6%)	0.691
Present	23(44.2%)	21 (40.4%)	
Vascular invasion			
Absent	27 (51.9%)	32(61.5%)	0.322
Present	25 (48.1%)	20(38.5%)	
Distant metastasis			
Absent	49(94.2%)	50(96.2%)	0.663
Present	3(5.8%)	2(3.8%)	
Clinical stage			
I, II	29 (55.8%)	31 (59.6%)	0.691
III, IV	23(44.2%)	21 (40.4%)	

Supplementary Table 4. Correlation between PFKFB3 expression and clinicopathological features in 104 ESCC patients.

The *P* value was determined by a Chi-square test. All the statistical tests were two-sided. Abbreviations: m: tumor invasion of mucosa; sm: submocosa; mp: muscularis propria; ss: subserosa; se: serosa penetration; si: invasion to adjacent structures.

Supplementary Table 5. Correlation between *AGPG* /PFKFB3 expression and clinicopathological features in 104 ESCC patients

Characteristics	AGPG/ PFKFB3-Low (n=42)	Intermediate (n=21)	AGPG/ PFKFB3- High (n=41)	P value
Age				
<58	21 (50.0%)	15(71.4%)	23(56.1%)	0.268
≥58	21 (50.0%)	6(28.6%)	18(43.9%)	
Gender				
Female	12 (28.6%)	7(33.3%)	12(29.3%)	0.923
Male	30 (71.4%)	14 (66.7%)	29(70.7%)	
Differentiation status				
Well/Moderate	37 (88.1%)	17 (81.0%)	34(82.9%)	0.869
Poor and others	5(11.9%)	4(19.0%)	7(17.1%)	
Tumor depth				
m/sm/mp	17 (40.5%)	6(28.6%)	10(24.4%)	0.236
ss/se/si	24 (59.5%)	15 (71.4%)	31(75.6%)	
Lymph node invasion				
Absent	25 (59.5%)	11 (52.4%)	24(58.5%)	0.855
Present	17(40.5%)	10 (47.6%)	17(41.5%)	
Vascular invasion				
Absent	23 (54.8%)	11(52.4%)	25(61.0%)	0.768
Present	19(45.2%)	10(47.6%)	16(39.0%)	
Distant metastasis				
Absent	40(95.2%)	20(95.2%)	39(95.1%)	0.998
Present	2(4.8%)	1(4.8%)	2(4.9%)	
Clinical stage				
I, II	25 (59.5%)	11 (52.4%)	24(58.5%)	0.855
III, IV	17(40.5%)	10 (47.6%)	17(41.5%)	

Supplementary Table 5. Correlation between *AGPG* /PFKFB3 expression and clinicopathological features in 104 ESCC patients. The *P* value was determined by a Chi-square test. All the statistical tests were two-sided. Abbreviations: m: tumor invasion of mucosa; sm: submocosa; mp: muscularis propria; ss: subserosa; se: serosa penetration; si: invasion to adjacent structures.

Supplementary Table 6. Sequence of siRNA, shRNA or sgRNA used in this study

Name	Sequence
ASO *AGPG*	TTCACCACCACAGCCAAACG
si *p53*	AACTACTTCCTGAAAACAA
sh *AGPG* #1	TTCACCACCACAGCCAAACG
sh *AGPG* #2	GACTTTGTTAACCGTTCCCT
AGPG sgRNA01	CGGCGGGGCTGTTTCGTAAG
AGPG sgRNA02	ATCAAGTGTCCTATATGCGT

Supplementary Table 7. Sequence of primers used in q-PCR analysis

Name	Forward primer	Reverse primer
β-Actin	CATGTACGTTGCTATCCAGGC	CTCCTTAATGTCACGCACGAT
U6	GCTTCGGCAGCACATATACTAA	TTGCGTGTCATCCTTGCG
p53	CAGCACATGACGGAGGTTGT	TCATCCAAATACTCCACACGC
AGPG	GGAGGCGGAGGTTGTAGTGAA	TTGTGGGAGGGAAGTCTGTGG
PFKFB3	TTGGCGTCCCCACAAAAGT	AGTTGTAGGAGCTGTACTGCTT
AGPG-promoter 922-939	CAAAGTGCTGGGATTATAGGCG	CTTGACTCCACTTTTCTCCTGTTTG
