# Supplementary material for: Long noncoding RNA AGPG regulates PFKFB3-mediated tumor glycolytic reprogramming
Source: Nat Commun. 2020 Mar 20;11:1507. doi: 10.1038/s41467-020-15112-3 (PMC7083971; doi:10.1038/s41467-020-15112-3)
Supplement: Supplementary file 3 — Description of Additional Supplementary Files [file 41467_2020_15112_MOESM3_ESM.pdf]

Description of Additional Supplementary Files

File Name: Supplementary Data 1
Description: List of candidate AGPG binding proteins from MS analysis

File Name: Supplementary Data 2
Description: List of antisense AGPG binding proteins from the MS analysis.
