# Supplementary material for: Long noncoding RNA AGPG regulates PFKFB3-mediated tumor glycolytic reprogramming
Source: Nat Commun. 2020 Mar 20;11:1507. doi: 10.1038/s41467-020-15112-3 (PMC7083971; doi:10.1038/s41467-020-15112-3)
Supplement: Supplementary file 4 — Supplementary Data 1 [file 41467_2020_15112_MOESM4_ESM.pdf]

Supplementary Data 1. List of candidate *AGPG* binding proteins from MS analysis

num	prot_desc	score	mass	matches	sequences	cover	len	emPAI
1	Keratin, type II cytoskeletal 1 OS=Homo sapiens GN=KRT1 PE=1 SV=6	999	65999	51	27	42.2	644	4.21
2	Actin, cytoplasmic 1 OS=Homo sapiens GN=ACTB PE=1 SV=1	730	41710	36	14	45.9	375	3.96
3	Keratin, type I cytoskeletal 9 OS=Homo sapiens GN=KRT9 PE=1 SV=3	577	62027	24	16	40.8	623	1.96
4	Keratin, type II cytoskeletal 2 epidermal OS=Homo sapiens GN=KRT2 PE=1 SV=2	517	65393	23	17	34.7	639	1.42
5	Keratin, type I cytoskeletal 10 OS=Homo sapiens GN=KRT10 PE=1 SV=6	506	58792	27	18	26.2	584	2.14
6	Alpha-enolase OS=Homo sapiens GN=ENO1 PE=1 SV=2	491	47139	26	14	34.8	434	1.76
7	Heat shock cognate 71 kDa protein OS=Homo sapiens GN=HSPA8 PE=1 SV=1	359	70854	19	13	24.3	646	1.06
8	Glyceraldehyde-3-phosphate dehydrogenase OS=Homo sapiens GN=GAPDH PE=1 SV=3	333	36030	28	9	38.8	335	1.63
9	Beta-actin-like protein 2 OS=Homo sapiens GN=ACTBL2 PE=1 SV=2	315	41976	13	4	13.8	376	0.35
10	Annexin A2 OS=Homo sapiens GN=ANXA2 PE=1 SV=2	297	38580	19	12	42.2	339	1.47
11	Heat shock protein HSP 90-beta OS=Homo sapiens GN=HSP90AB1 PE=1 SV=4	239	83212	19	15	22.4	724	0.65
12	Gamma-enolase OS=Homo sapiens GN=ENO2 PE=1 SV=3	225	47239	6	2	7.1	434	0.22
13	Heat shock protein HSP 90-alpha OS=Homo sapiens GN=HSP90AA1 PE=1 SV=5	215	84607	17	15	21	732	0.58
14	6-phosphofructo-2-kinase/fructose-2,6-bisphosphatase 3 OS=Homo sapiens GN=PFKFB3 PE=1 SV=1	213	72288	11	10	18.8	520	0.49
15	ATP synthase subunit alpha, mitochondrial OS=Homo sapiens GN=ATP5A1 PE=1 SV=1	204	59714	11	3	4.9	553	0.17
16	Peroxiredoxin-1 OS=Homo sapiens GN=PRDX1 PE=1 SV=1	203	22096	10	5	21.1	199	1.03
17	Annexin A1 OS=Homo sapiens GN=ANXA1 PE=1 SV=2	168	38690	5	4	15.6	346	0.39
18	14-3-3 protein zeta/delta OS=Homo sapiens GN=YWHAZ PE=1 SV=1	159	27728	6	4	17.6	245	0.41
19	Elongation factor 1-alpha 2 OS=Homo sapiens GN=EEF1A2 PE=1 SV=1	155	50438	10	8	20.3	463	0.77
20	Fructose-bisphosphate aldolase A OS=Homo sapiens GN=ALDOA PE=1 SV=2	154	39395	7	6	22.8	364	0.62
21	Keratin, type I cytoskeletal 16 OS=Homo sapiens GN=KRT16 PE=1 SV=4	153	51236	12	11	17.1	473	0.65
22	Prelamin-A/C OS=Homo sapiens GN=LMNA PE=1 SV=1	148	74095	14	13	17.2	664	0.41
23	Heat shock 70 kDa protein 1A OS=Homo sapiens GN=HSPA1A PE=1 SV=1	145	70009	5	5	10	641	0.2
24	Heat shock 70 kDa protein 6 OS=Homo sapiens GN=HSPA6 PE=1 SV=2	141	70984	4	4	7.5	643	0.15
25	40S ribosomal protein SA OS=Homo sapiens GN=RPSA PE=1 SV=4	133	32833	5	4	18	295	0.62
26	Brain acid soluble protein 1 OS=Homo sapiens GN=BASP1 PE=1 SV=2	132	22680	3	3	21.6	227	0.51
27	Serum albumin OS=Homo sapiens GN=ALB PE=1 SV=2	131	69321	12	8	12.3	609	0.26
28	APC membrane recruitment protein 1 OS=Homo sapiens GN=AMER1 PE=1 SV=2	130	123952	25	1	0.9	1135	0.03
29	Nucleophosmin OS=Homo sapiens GN=NPM1 PE=1 SV=2	128	32555	7	5	25.9	294	0.48
30	Keratin, type II cytoskeletal 5 OS=Homo sapiens GN=KRT5 PE=1 SV=3	127	62340	8	7	11.2	590	0.43
31	Stress-70 protein, mitochondrial OS=Homo sapiens GN=HSPA9 PE=1 SV=2	124	73635	6	6	11.3	679	0.24
32	Peroxiredoxin-4 OS=Homo sapiens GN=PRDX4 PE=1 SV=1	120	30521	6	3	8.5	271	0.23
33	Keratin, type I cytoskeletal 14 OS=Homo sapiens GN=KRT14 PE=1 SV=4	119	51529	13	11	17.2	472	0.54
34	Elongation factor 2 OS=Homo sapiens GN=EEF2 PE=1 SV=4	117	95277	9	9	9.9	858	0.27
35	Putative elongation factor 1-alpha-like 3 OS=Homo sapiens GN=EEF1A1P5 PE=5 SV=1	115	50153	9	7	15.2	462	0.56
36	Glutathione S-transferase P OS=Homo sapiens GN=GSTP1 PE=1 SV=2	111	23341	8	5	34.8	210	1.24
37	Electron transfer flavoprotein subunit alpha, mitochondrial OS=Homo sapiens GN=ETFA PE=1 SV=1	110	35058	7	2	7.8	333	0.44
38	Triosephosphate isomerase OS=Homo sapiens GN=TPI1 PE=1 SV=3	110	30772	6	5	22.4	286	0.67
39	Nucleolin OS=Homo sapiens GN=NCL PE=1 SV=3	106	76568	6	6	7.9	710	0.23
40	Glucose-6-phosphate isomerase OS=Homo sapiens GN=GPI PE=1 SV=4	100	63107	5	4	10.6	558	0.23
41	Endoplasmin OS=Homo sapiens GN=HSP90B1 PE=1 SV=1	100	92411	5	5	6.1	803	0.15
42	Keratin, type I cytoskeletal 17 OS=Homo sapiens GN=KRT17 PE=1 SV=2	99	48076	10	9	15	432	0.49
43	Creatine kinase B-type OS=Homo sapiens GN=CKB PE=1 SV=1	98	42617	4	3	12.1	381	0.25
44	L-lactate dehydrogenase A chain OS=Homo sapiens GN=LDHA PE=1 SV=2	98	36665	1	1	3	332	0.09
45	Keratin, type II cytoskeletal 6B OS=Homo sapiens GN=KRT6B PE=1 SV=5	96	60030	7	4	6.9	564	0.24
46	Keratin, type I cytoskeletal 19 OS=Homo sapiens GN=KRT19 PE=1 SV=4	93	44079	7	7	13.3	400	0.44
47	Calreticulin OS=Homo sapiens GN=CALR PE=1 SV=1	93	48112	6	4	9.8	417	0.3
48	Non-receptor tyrosine-protein kinase TYK2 OS=Homo sapiens GN=TYK2 PE=1 SV=3	93	133565	5	3	1.6	1187	0.05
49	14-3-3 protein gamma OS=Homo sapiens GN=YWHAG PE=1 SV=2	90	28285	3	3	12.1	247	0.25
50	Tyrosine-protein kinase JAK1 OS=Homo sapiens GN=JAK1 PE=1 SV=2	89	133191	3	1	0.5	1154	0.02
51	Profilin-1 OS=Homo sapiens GN=PFN1 PE=1 SV=2	89	15045	2	1	12.9	140	0.23
52	Tubulin beta chain OS=Homo sapiens GN=TUBB PE=1 SV=2	89	49639	4	3	9.2	444	0.21
53	60 kDa heat shock protein, mitochondrial OS=Homo sapiens GN=HSPD1 PE=1 SV=2	87	61016	5	5	9.4	573	0.23
54	14-3-3 protein theta OS=Homo sapiens GN=YWHAQ PE=1 SV=1	87	27747	4	4	15.9	245	0.25
55	GTP-binding nuclear protein Ran OS=Homo sapiens GN=RAN PE=1 SV=3	86	24408	4	4	19.4	216	0.67
56	Keratin, type II cytoskeletal 8 OS=Homo sapiens GN=KRT8 PE=1 SV=7	86	53671	6	5	8.7	483	0.27
57	Elongation factor 1-gamma OS=Homo sapiens GN=EEF1G PE=1 SV=3	85	50087	4	3	6.9	437	0.29
58	Keratin, type II cytoskeletal 6A OS=Homo sapiens GN=KRT6A PE=1 SV=3	82	60008	6	5	9.8	564	0.24
59	Transketolase OS=Homo sapiens GN=TKT PE=1 SV=3	82	67835	10	7	12.2	623	0.21
60	Keratin, type I cytoskeletal 13 OS=Homo sapiens GN=KRT13 PE=1 SV=4	82	49557	6	5	6.6	458	0.29
61	Dihydrolipoyllysine-residue succinyltransferase component of 2-oxoglutarate dehydrogenase complex	77	48724	4	2	4.6	453	0.07
62	Protein deglycase DJ-1 OS=Homo sapiens GN=PARK7 PE=1 SV=2	76	19878	2	2	12.2	189	0.17
63	Hornerin OS=Homo sapiens GN=HRNR PE=1 SV=2	76	282228	3	3	2	2850	0.04
64	Suprabasin OS=Homo sapiens GN=SBSN PE=1 SV=2	75	60505	3	3	8.5	590	0.11
65	Phosphatidylethanolamine-binding protein 1 OS=Homo sapiens GN=PEBP1 PE=1 SV=3	72	21044	4	4	43.3	187	0.56
66	Fascin OS=Homo sapiens GN=FSCN1 PE=1 SV=3	72	54496	2	2	5.3	493	0.12
67	Splicing factor, proline- and glutamine-rich OS=Homo sapiens GN=SFPQ PE=1 SV=2	71	76102	5	5	6.1	707	0.13
68	Peroxiredoxin-2 OS=Homo sapiens GN=PRDX2 PE=1 SV=5	70	21878	3	2	14.6	198	0.33
69	Protein disulfide-isomerase A3 OS=Homo sapiens GN=PDIA3 PE=1 SV=4	69	56747	9	8	18	505	0.33
70	Moesin OS=Homo sapiens GN=MSN PE=1 SV=3	68	67778	5	4	7.3	577	0.21
71	Nucleoside diphosphate kinase A OS=Homo sapiens GN=NME1 PE=1 SV=1	67	17138	3	2	19.1	152	0.43
72	Nuclear migration protein nudC OS=Homo sapiens GN=NUDC PE=1 SV=1	63	38219	2	2	6	331	0.18
73	Argininosuccinate synthase OS=Homo sapiens GN=ASS1 PE=1 SV=2	63	46501	3	3	5.6	412	0.23
74	Keratin, type I cytoskeletal 18 OS=Homo sapiens GN=KRT18 PE=1 SV=2	62	48029	5	5	8.1	430	0.3
75	Chloride intracellular channel protein 1 OS=Homo sapiens GN=CLIC1 PE=1 SV=4	62	26906	2	2	12.4	241	0.26
76	Ras GTPase-activating-like protein IQGAP1 OS=Homo sapiens GN=IQGAP1 PE=1 SV=1	61	189134	3	3	2	1657	0.05
77	Heterogeneous nuclear ribonucleoproteins A2/B1 OS=Homo sapiens GN=HNRNPA2B1 PE=1 SV=2	61	37407	2	2	6.2	353	0.18
78	ATP synthase subunit beta, mitochondrial OS=Homo sapiens GN=ATP5B PE=1 SV=3	61	56525	3	2	5.7	529	0.12
79	Heat shock protein 75 kDa, mitochondrial OS=Homo sapiens GN=TRAP1 PE=1 SV=3	59	80060	1	1	2	704	0.04
80	Transgelin-2 OS=Homo sapiens GN=TAGLN2 PE=1 SV=3	59	22377	3	3	18.6	199	0.52
81	Adenosylhomocysteinase OS=Homo sapiens GN=AHCY PE=1 SV=4	59	47685	2	2	4.4	432	0.14
82	Vacuolar protein sorting-associated protein 13D OS=Homo sapiens GN=VPS13D PE=1 SV=2	58	491606	3	1	0.2	4388	0.01

83	Chloride intracellular channel protein 4 OS=Homo sapiens GN=CLIC4 PE=1 SV=4	58	28754	1	1	4.7	253	0.12
84	Tyrosine-protein kinase JAK3 OS=Homo sapiens GN=JAK3 PE=1 SV=2	58	125019	3	1	0.5	1124	0.03
85	rine/threonine-protein phosphatase PP1-beta catalytic subunit OS=Homo sapiens GN=PPP1CB PE=1 SV	57	37163	1	1	3.1	327	0.09
86	Spectrin beta chain, non-erythrocytic 5 OS=Homo sapiens GN=SPTBN5 PE=1 SV=2	57	416493	7	5	0.9	3674	0.01
87	PCNA-interacting partner OS=Homo sapiens GN=PARPBP PE=1 SV=3	57	65013	3	1	1	579	0.05
88	Maestro heat-like repeat-containing protein family member 7 OS=Homo sapiens GN=MROH7 PE=2 SV=4	57	145554	3	1	0.5	1323	0.02
89	Urokinase-type plasminogen activator OS=Homo sapiens GN=PLAU PE=1 SV=2	57	48476	3	1	1.4	431	0.07
90	Proteasome subunit beta type-4 OS=Homo sapiens GN=PSMB4 PE=1 SV=4	57	29185	1	1	3.8	264	0.11
91	Lysozyme C OS=Homo sapiens GN=LYZ PE=1 SV=1	56	16526	2	1	8.1	148	0.45
92	Inorganic pyrophosphatase OS=Homo sapiens GN=PPA1 PE=1 SV=2	55	32639	2	2	10.7	289	0.21
93	Radixin OS=Homo sapiens GN=RDX PE=1 SV=1	54	68521	3	2	3.3	583	0.1
94	Eukaryotic translation initiation factor 2 subunit 1 OS=Homo sapiens GN=EIF2S1 PE=1 SV=3	54	36089	3	3	7.9	315	0.19
95	Hsp90 co-chaperone Cdc37 OS=Homo sapiens GN=CDC37 PE=1 SV=1	53	44440	2	1	2.4	378	0.07
96	Malate dehydrogenase, mitochondrial OS=Homo sapiens GN=MDH2 PE=1 SV=3	53	35481	5	4	17.2	338	0.31
97	Tropomyosin alpha-3 chain OS=Homo sapiens GN=TPM3 PE=1 SV=2	53	32930	3	3	13	285	0.33
98	Leucine-rich repeat-containing protein 9 OS=Homo sapiens GN=LRRC9 PE=2 SV=2	52	166805	1	1	0.5	1453	0.02
99	Phosphoglycerate kinase 1 OS=Homo sapiens GN=PGK1 PE=1 SV=3	52	44586	7	6	17.5	417	0.24
100	Tubulin alpha-1C chain OS=Homo sapiens GN=TUBA1C PE=1 SV=1	52	49863	3	3	10	449	0.14
101	Serine--tRNA ligase, mitochondrial OS=Homo sapiens GN=SARS2 PE=1 SV=1	52	58246	3	1	1.7	518	0.06
102	Heterogeneous nuclear ribonucleoprotein D0 OS=Homo sapiens GN=HNRNPD PE=1 SV=1	52	38410	1	1	2	355	0.09
103	14-3-3 protein beta/alpha OS=Homo sapiens GN=YWHAB PE=1 SV=3	51	28065	5	4	15.9	246	0.4
104	lependent Clp protease ATP-binding subunit clpX-like, mitochondrial OS=Homo sapiens GN=CLPX PE=1	51	69181	1	1	2.1	633	0.05
105	Aspartate aminotransferase, mitochondrial OS=Homo sapiens GN=GOT2 PE=1 SV=3	50	47487	2	1	3.3	430	0.07
106	Otoancorin OS=Homo sapiens GN=OTOA PE=1 SV=1	50	128451	1	1	0.5	1153	0.03
107	Heterogeneous nuclear ribonucleoprotein A1 OS=Homo sapiens GN=HNRNPA1 PE=1 SV=5	50	38723	2	2	5.6	372	0.18
108	Keratin, type I cytoskeletal 27 OS=Homo sapiens GN=KRT27 PE=1 SV=2	48	49792	4	3	5.9	459	0.14
109	Alpha-actinin-1 OS=Homo sapiens GN=ACTN1 PE=1 SV=2	48	102993	2	1	1.3	892	0.03
110	Proteasome subunit alpha type-6 OS=Homo sapiens GN=PSMA6 PE=1 SV=1	48	27382	1	1	5.3	246	0.12
111	Synemin OS=Homo sapiens GN=SYNM PE=1 SV=2	47	172663	2	2	1	1565	0.02
112	HLA class I histocompatibility antigen, B-14 alpha chain OS=Homo sapiens GN=HLA-B PE=1 SV=1	47	40333	4	4	12.2	362	0.27
113	Pyruvate kinase PKM OS=Homo sapiens GN=PKM PE=1 SV=4	47	57900	1	1	3	531	0.06
114	Prohibitin-2 OS=Homo sapiens GN=PHB2 PE=1 SV=2	47	33276	3	3	12.4	299	0.1
115	Dermcidin OS=Homo sapiens GN=DCD PE=1 SV=2	46	11277	2	2	22.7	110	0.7
116	Polycystic kidney disease protein 1-like 2 OS=Homo sapiens GN=PKD1L2 PE=1 SV=4	46	272402	1	1	0.3	2459	0.01
117	Cytochrome c oxidase subunit 7A2, mitochondrial OS=Homo sapiens GN=COX7A2 PE=1 SV=1	46	9390	2	1	7.2	83	0.38
118	Non-POU domain-containing octamer-binding protein OS=Homo sapiens GN=NONO PE=1 SV=4	45	54197	2	2	4.2	471	0.06
119	Serine/arginine-rich splicing factor 11 OS=Homo sapiens GN=SRSF11 PE=1 SV=1	45	53510	2	1	1.7	484	0.06
120	Transaldolase OS=Homo sapiens GN=TALDO1 PE=1 SV=2	45	37516	1	1	3.3	337	0.09
121	Alpha-actinin-4 OS=Homo sapiens GN=ACTN4 PE=1 SV=2	45	104788	4	3	4.2	911	0.06
122	Heterogeneous nuclear ribonucleoprotein K OS=Homo sapiens GN=HNRNPK PE=1 SV=1	45	50944	4	3	8.2	463	0.13
123	Phosphoglycerate mutase 1 OS=Homo sapiens GN=PGAM1 PE=1 SV=2	44	28786	4	3	15.7	254	0.39
124	Putative glycerol kinase 3 OS=Homo sapiens GN=GK3P PE=5 SV=2	44	60559	2	2	2.7	553	0.05
125	Vinculin OS=Homo sapiens GN=VCL PE=1 SV=4	44	123722	2	2	1.7	1134	0.03
126	EMILIN-1 OS=Homo sapiens GN=EMILIN1 PE=1 SV=2	43	106601	1	1	0.7	1016	0.03
127	Elongation factor 1-delta OS=Homo sapiens GN=EEF1D PE=1 SV=5	43	31103	1	1	8.5	281	0.11
128	Voltage-dependent anion-selective channel protein 2 OS=Homo sapiens GN=VDAC2 PE=1 SV=2	42	31547	1	1	6.8	294	0.11
129	DNA topoisomerase 2-beta OS=Homo sapiens GN=TOP2B PE=1 SV=3	42	183152	1	1	0.4	1626	0.02
130	Cytochrome c oxidase subunit 5A, mitochondrial OS=Homo sapiens GN=COX5A PE=1 SV=2	42	16752	1	1	4	150	0.2
131	Heterogeneous nuclear ribonucleoprotein U OS=Homo sapiens GN=HNRNPU PE=1 SV=6	42	90528	2	2	2.7	825	0.04
132	Protein disulfide-isomerase A4 OS=Homo sapiens GN=PDIA4 PE=1 SV=2	42	72887	3	3	5.3	645	0.05
133	Myotubularin-related protein 11 OS=Homo sapiens GN=MTMR11 PE=2 SV=2	42	79495	1	1	1	709	0.04
134	Putative lipocalin 1-like protein 1 OS=Homo sapiens GN=LCN1P1 PE=5 SV=1	42	17907	1	1	6.8	162	0.19
135	Fibrous sheath-interacting protein 2 OS=Homo sapiens GN=FSIP2 PE=2 SV=4	42	780119	5	3	0.4	6907	0.01
136	Calpain small subunit 1 OS=Homo sapiens GN=CAPNS1 PE=1 SV=1	41	28298	1	1	5.6	268	0.12
137	Heterogeneous nuclear ribonucleoprotein A3 OS=Homo sapiens GN=HNRNPA3 PE=1 SV=2	41	39571	2	2	6.6	378	0.17
138	Heat shock protein beta-1 OS=Homo sapiens GN=HSPB1 PE=1 SV=2	41	22768	1	1	8.3	205	0.15
139	Ankyrin repeat domain-containing protein SOWAHA OS=Homo sapiens GN=SOWAHA PE=1 SV=3	41	57407	1	1	1.5	549	0.06
140	X-ray repair cross-complementing protein 5 OS=Homo sapiens GN=XRCC5 PE=1 SV=3	41	82652	1	1	1.9	732	0.04
141	DNA methyltransferase 1-associated protein 1 OS=Homo sapiens GN=DMAP1 PE=1 SV=1	40	52960	2	1	1.7	467	0.06
142	Alkaline phosphatase, placental type OS=Homo sapiens GN=ALPP PE=1 SV=2	40	57917	2	2	6.4	535	0.06
143	HLA class I histocompatibility antigen, Cw-7 alpha chain OS=Homo sapiens GN=HLA-C PE=1 SV=3	40	40623	2	2	6.8	366	0.17
144	Plasmalemma vesicle-associated protein OS=Homo sapiens GN=PLVAP PE=2 SV=1	40	50562	2	2	3.2	442	0.07
145	P-selectin glycoprotein ligand 1 OS=Homo sapiens GN=SELPLG PE=1 SV=1	40	43174	1	1	1.9	412	0.08
146	Guanine deaminase OS=Homo sapiens GN=GDA PE=1 SV=1	40	50971	1	1	2.9	454	0.06
147	Nuclear GTPase SLIP-GC OS=Homo sapiens GN=NUGGC PE=2 SV=3	40	91074	1	1	0.9	796	0.04
148	T-complex protein 1 subunit gamma OS=Homo sapiens GN=CCT3 PE=1 SV=4	40	60495	1	1	1.3	545	0.05
149	Vacuolar protein sorting-associated protein 53 homolog OS=Homo sapiens GN=VPS53 PE=1 SV=1	40	79602	1	1	0.9	699	0.04
150	ccinyl-CoA:3-ketoacid coenzyme A transferase 1, mitochondrial OS=Homo sapiens GN=OXCT1 PE=1 SV	39	56122	2	2	3.5	520	0.06
151	Ribonuclease inhibitor OS=Homo sapiens GN=RNH1 PE=1 SV=2	39	49941	1	1	2.4	461	0.07
152	Liprin-beta-1 OS=Homo sapiens GN=PPFIBP1 PE=1 SV=2	39	113952	1	1	0.6	1011	0.03
153	Fumarate hydratase, mitochondrial OS=Homo sapiens GN=FH PE=1 SV=3	39	54602	1	1	3.5	510	0.06
154	Eukaryotic translation initiation factor 4 gamma 1 OS=Homo sapiens GN=EIF4G1 PE=1 SV=4	39	175382	2	2	0.9	1599	0.02
155	TFIIH basal transcription factor complex helicase XPD subunit OS=Homo sapiens GN=ERCC2 PE=1 SV=	39	86854	2	2	1.7	760	0.08
156	Fructose-bisphosphate aldolase C OS=Homo sapiens GN=ALDOC PE=1 SV=2	39	39431	1	1	7.1	364	0.08
157	Cofilin-2 OS=Homo sapiens GN=CFL2 PE=1 SV=1	39	18725	1	1	6.6	166	0.18
158	Histone-lysine N-methyltransferase SETD2 OS=Homo sapiens GN=SETD2 PE=1 SV=3	39	287418	1	1	0.3	2564	0.01
159	Ezrin OS=Homo sapiens GN=EZR PE=1 SV=4	39	69370	4	3	6.3	586	0.1
160	Desmoplakin OS=Homo sapiens GN=DSP PE=1 SV=3	39	331569	2	2	0.6	2871	0.01
161	Adenylate kinase 2, mitochondrial OS=Homo sapiens GN=AK2 PE=1 SV=2	38	26461	1	1	10.5	239	0.13
162	Peroxiredoxin-6 OS=Homo sapiens GN=PRDX6 PE=1 SV=3	38	25019	1	1	3.1	224	0.13
163	Ankyrin repeat and SOCS box protein 6 OS=Homo sapiens GN=ASB6 PE=1 SV=1	37	47106	1	1	1.7	421	0.07
164	14-3-3 protein sigma OS=Homo sapiens GN=SFN PE=1 SV=1	37	27757	2	2	6.5	248	0.12
165	T-complex protein 1 subunit zeta OS=Homo sapiens GN=CCT6A PE=1 SV=3	37	57988	2	2	5.3	531	0.12
166	DNA replication licensing factor MCM5 OS=Homo sapiens GN=MCM5 PE=1 SV=5	36	82233	5	2	1.9	734	0.04

167	Polyserase-2 OS=Homo sapiens GN=PRSS36 PE=1 SV=2	36	91896	1	1	0.7	855	0.04
168	Serine/threonine-protein kinase DCLK2 OS=Homo sapiens GN=DCLK2 PE=2 SV=4	36	83554	1	1	0.8	766	0.04
169	Enhancer of mRNA-decapping protein 4 OS=Homo sapiens GN=EDC4 PE=1 SV=1	36	151567	1	1	0.6	1401	0.02
170	Pleckstrin homology domain-containing family G member 3 OS=Homo sapiens GN=PLEKHG3 PE=1 SV=1	36	134329	1	1	0.6	1219	0.02
171	Sarcoplasmic/endoplasmic reticulum calcium ATPase 2 OS=Homo sapiens GN=ATP2A2 PE=1 SV=1	36	114683	1	1	0.6	1042	0.03
172	Charged multivesicular body protein 5 OS=Homo sapiens GN=CHMP5 PE=1 SV=1	36	24555	1	1	3.2	219	0.14
173	P with SH3 domain, ANK repeat and PH domain-containing protein 2 OS=Homo sapiens GN=ASAP2 PE=	36	111581	1	1	0.8	1006	0.03
174	Annexin A5 OS=Homo sapiens GN=ANXA5 PE=1 SV=2	36	35914	2	2	6.6	320	0.19
175	SKI family transcriptional corepressor 1 OS=Homo sapiens GN=SKOR1 PE=1 SV=1	36	99768	1	1	1	965	0.03
176	Voltage-dependent anion-selective channel protein 1 OS=Homo sapiens GN=VDAC1 PE=1 SV=2	36	30754	2	2	8.5	283	0.23
177	Catenin delta-2 OS=Homo sapiens GN=CTNND2 PE=1 SV=3	36	132574	1	1	0.5	1225	0.02
178	FGFR1 oncogene partner OS=Homo sapiens GN=FGFR1OP PE=1 SV=1	36	43039	1	1	1.5	399	0.08
179	A-kinase anchor protein 9 OS=Homo sapiens GN=AKAP9 PE=1 SV=3	36	453387	1	1	0.2	3911	0.01
180	Protein SETSIP OS=Homo sapiens GN=SETSIP PE=1 SV=1	36	34861	3	1	4.3	302	0.1
181	Rho GTPase-activating protein SYDE2 OS=Homo sapiens GN=SYDE2 PE=1 SV=2	36	133146	1	1	0.5	1194	0.02
182	Low-density lipoprotein receptor-related protein 1B OS=Homo sapiens GN=LRP1B PE=1 SV=2	36	515159	1	1	0.1	4599	0.01
183	Probable Xaa-Pro aminopeptidase 3 OS=Homo sapiens GN=XPNPEP3 PE=1 SV=1	35	56997	1	1	1.4	507	0.06
184	Bromodomain and WD repeat-containing protein 1 OS=Homo sapiens GN=BRWD1 PE=1 SV=4	35	262772	1	1	0.3	2320	0.01
185	Insulin receptor OS=Homo sapiens GN=INSR PE=1 SV=4	35	156232	2	2	0.9	1382	0.04
186	4F2 cell-surface antigen heavy chain OS=Homo sapiens GN=SLC3A2 PE=1 SV=3	35	67952	2	1	1.9	630	0.1
187	Rho GTPase-activating protein 11B OS=Homo sapiens GN=ARHGAP11B PE=2 SV=1	35	30232	1	1	2.2	267	0.11
188	Myosin light chain kinase family member 4 OS=Homo sapiens GN=MYLK4 PE=1 SV=2	35	44480	1	1	2.3	388	0.07
189	Histone-binding protein RBBP4 OS=Homo sapiens GN=RBBP4 PE=1 SV=3	35	47626	1	1	1.6	425	0.07
190	Embryonic polyadenylate-binding protein 2 OS=Homo sapiens GN=PABPN1L PE=2 SV=1	35	30367	2	1	2.5	278	0.11
191	HLA class I histocompatibility antigen, alpha chain G OS=Homo sapiens GN=HLA-G PE=1 SV=1	34	38200	2	2	6.8	338	0.09
192	N6-adenosine-methyltransferase 70 kDa subunit OS=Homo sapiens GN=METTL3 PE=1 SV=2	34	64433	1	1	1.4	580	0.05
193	Peroxisome biogenesis factor 1 OS=Homo sapiens GN=PEX1 PE=1 SV=1	34	142778	1	1	0.7	1283	0.02
194	ve selection and upkeep of intraepithelial T-cells protein 1 homolog OS=Homo sapiens GN=SKINTL PE=5	34	25389	1	1	6	218	0.13
195	Protein Spindly OS=Homo sapiens GN=SPDL1 PE=1 SV=2	34	70128	1	1	1.5	605	0.05
196	Centrosome-associated protein 350 OS=Homo sapiens GN=CEP350 PE=1 SV=1	34	350716	1	1	0.2	3117	0.01
