# Supplementary material for: Long noncoding RNA AGPG regulates PFKFB3-mediated tumor glycolytic reprogramming
Source: Nat Commun. 2020 Mar 20;11:1507. doi: 10.1038/s41467-020-15112-3 (PMC7083971; doi:10.1038/s41467-020-15112-3)
Supplement: Supplementary file 5 — Supplementary Data 2 [file 41467_2020_15112_MOESM5_ESM.pdf]

Supplementary Data 2. List of antisense AGPG binding proteins from the MS analysis.

num	prot_desc	score	mass	matches	sequences	cover	len	emPAI
1	Actin, cytoplasmic 1 OS=Homo sapiens GN=ACTB PE=1 SV=1	831	41710	40	17	50.9	375	5.72
2	Keratin, type II cytoskeletal 1 OS=Homo sapiens GN=KRT1 PE=1 SV=6	827	65999	37	23	42.5	644	2.21
3	Glyceraldehyde-3-phosphate dehydrogenase OS=Homo sapiens GN=GAPDH PE=1 SV=3	693	36030	52	14	48.7	335	8.83
4	Alpha-enolase OS=Homo sapiens GN=ENO1 PE=1 SV=2	673	47139	31	13	36.2	434	1.58
5	Heat shock protein HSP 90-alpha OS=Homo sapiens GN=HSP90AA1 PE=1 SV=5	646	84607	38	17	25.3	732	1.31
6	Heat shock cognate 71 kDa protein OS=Homo sapiens GN=HSPA8 PE=1 SV=1	579	70854	28	15	25.2	646	1.26
7	Heat shock protein HSP 90-beta OS=Homo sapiens GN=HSP90AB1 PE=1 SV=4	563	83212	30	20	29.7	724	1.34
8	Actin, alpha cardiac muscle 1 OS=Homo sapiens GN=ACTC1 PE=1 SV=1	556	41992	27	12	30.2	377	2.12
9	Keratin, type I cytoskeletal 9 OS=Homo sapiens GN=KRT9 PE=1 SV=3	526	62027	17	14	35.3	623	1.28
10	Keratin, type I cytoskeletal 10 OS=Homo sapiens GN=KRT10 PE=1 SV=6	337	58792	19	14	26.2	584	1.26
11	Ubiquitin-like modifier-activating enzyme 1 OS=Homo sapiens GN=UBA1 PE=1 SV=3	325	117774	19	12	16.8	1058	0.35
12	Heat shock 70 kDa protein 1A OS=Homo sapiens GN=HSPA1A PE=1 SV=1	311	70009	9	6	11.1	641	0.26
13	78 kDa glucose-regulated protein OS=Homo sapiens GN=HSPA5 PE=1 SV=2	309	72288	16	12	24	654	0.7
14	Prelamin-A/C OS=Homo sapiens GN=LMNA PE=1 SV=1	306	74095	22	16	24.8	664	0.83
15	Heat shock 70 kDa protein 6 OS=Homo sapiens GN=HSPA6 PE=1 SV=2	305	70984	7	4	7.6	643	0.2
16	Annexin A2 OS=Homo sapiens GN=ANXA2 PE=1 SV=2	304	38580	20	17	51.9	339	1.91
17	Glutathione S-transferase P OS=Homo sapiens GN=GSTP1 PE=1 SV=2	296	23341	12	7	55.7	210	2.35
18	Keratin, type II cytoskeletal 8 OS=Homo sapiens GN=KRT8 PE=1 SV=7	293	53671	16	11	22.8	483	0.61
19	Beta-enolase OS=Homo sapiens GN=ENO3 PE=1 SV=5	290	46957	9	3	11.5	434	0.07
20	Beta-actin-like protein 2 OS=Homo sapiens GN=ACTBL2 PE=1 SV=2	285	41976	15	6	14.6	376	0.58
21	40S ribosomal protein SA OS=Homo sapiens GN=RPSA PE=1 SV=4	283	32833	7	2	10.2	295	0.21
22	Phosphoglycerate kinase 1 OS=Homo sapiens GN=PGK1 PE=1 SV=3	280	44586	18	7	27.3	417	1.19
23	Elongation factor 1-alpha 2 OS=Homo sapiens GN=EEF1A2 PE=1 SV=1	276	50438	16	7	18.4	463	0.66
24	Vinculin OS=Homo sapiens GN=VCL PE=1 SV=4	275	123722	15	12	14	1134	0.33
25	Elongation factor 2 OS=Homo sapiens GN=EEF2 PE=1 SV=4	270	95277	15	7	10.6	858	0.31
26	Keratin, type II cytoskeletal 2 epidermal OS=Homo sapiens GN=KRT2 PE=1 SV=2	269	65393	17	16	38	639	0.89
27	Putative elongation factor 1-alpha-like 3 OS=Homo sapiens GN=EEF1A1P5 PE=5 SV=1	247	50153	17	8	19.9	462	0.56
28	ATP synthase subunit beta, mitochondrial OS=Homo sapiens GN=ATP5B PE=1 SV=3	244	56525	10	9	25.3	529	0.57
29	60 kDa heat shock protein, mitochondrial OS=Homo sapiens GN=HSPD1 PE=1 SV=2	223	61016	12	9	23.6	573	0.78
30	Malate dehydrogenase, mitochondrial OS=Homo sapiens GN=MDH2 PE=1 SV=3	213	35481	9	5	20.4	338	0.87
31	Peroxiredoxin-1 OS=Homo sapiens GN=PRDX1 PE=1 SV=1	211	22096	18	9	39.2	199	3.73
32	Keratin, type I cytoskeletal 18 OS=Homo sapiens GN=KRT18 PE=1 SV=2	211	48029	13	12	32.1	430	1.08
33	Transketolase OS=Homo sapiens GN=TKT PE=1 SV=3	203	67835	15	12	22.6	623	0.76
34	Creatine kinase B-type OS=Homo sapiens GN=CKB PE=1 SV=1	203	42617	8	5	18.4	381	0.45
35	Triosephosphate isomerase OS=Homo sapiens GN=TPI1 PE=1 SV=3	201	30772	20	8	31.8	286	1.52
36	Protein disulfide-isomerase A3 OS=Homo sapiens GN=PDIA3 PE=1 SV=4	199	56747	10	8	19.6	505	0.66
37	14-3-3 protein zeta/delta OS=Homo sapiens GN=YWHAZ PE=1 SV=1	189	27728	6	5	28.6	245	0.76
38	Fructose-bisphosphate aldolase A OS=Homo sapiens GN=ALDOA PE=1 SV=2	187	39395	12	10	31.6	364	0.91
39	Protein disulfide-isomerase OS=Homo sapiens GN=P4HB PE=1 SV=3	185	57081	11	9	22.2	508	0.66
40	Alpha-actinin-4 OS=Homo sapiens GN=ACTN4 PE=1 SV=2	178	104788	10	8	11.3	911	0.28
41	Endoplasmin OS=Homo sapiens GN=HSP90B1 PE=1 SV=1	175	92411	9	7	11.2	803	0.23
42	Serum albumin OS=Homo sapiens GN=ALB PE=1 SV=2	175	69321	10	5	6.4	609	0.15
43	Protein disulfide-isomerase A4 OS=Homo sapiens GN=PDIA4 PE=1 SV=2	175	72887	6	4	8.7	645	0.25
44	Pyruvate kinase PKM OS=Homo sapiens GN=PKM PE=1 SV=4	174	57900	9	6	16.2	531	0.39
45	Nucleoside diphosphate kinase A OS=Homo sapiens GN=NME1 PE=1 SV=1	173	17138	10	2	19.1	152	1.06
46	14-3-3 protein sigma OS=Homo sapiens GN=SFN PE=1 SV=1	169	27757	5	3	12.9	248	0.57
47	Moesin OS=Homo sapiens GN=MSN PE=1 SV=3	168	67778	16	13	18.9	577	0.53
48	Brain acid soluble protein 1 OS=Homo sapiens GN=BASP1 PE=1 SV=2	167	22680	6	6	40.1	227	0.74
49	Annexin A1 OS=Homo sapiens GN=ANXA1 PE=1 SV=2	163	38690	5	5	17.1	346	0.39
50	Heterogeneous nuclear ribonucleoprotein K OS=Homo sapiens GN=HNRNPK PE=1 SV=1	148	50944	5	4	12.1	463	0.28
51	Alpha-actinin-1 OS=Homo sapiens GN=ACTN1 PE=1 SV=2	130	102993	7	6	7.7	892	0.17
52	Calreticulin OS=Homo sapiens GN=CALR PE=1 SV=1	127	48112	5	4	12.5	417	0.39
53	Glucose-6-phosphate isomerase OS=Homo sapiens GN=GPI PE=1 SV=4	126	63107	6	4	8.4	558	0.16
54	Prohibitin-2 OS=Homo sapiens GN=PHB2 PE=1 SV=2	122	33276	5	4	17.4	299	0.46
55	Transgelin-2 OS=Homo sapiens GN=TAGLN2 PE=1 SV=3	121	22377	5	4	24.1	199	0.75
56	Nucleolin OS=Homo sapiens GN=NCL PE=1 SV=3	120	76568	6	6	11	710	0.18
57	14-3-3 protein gamma OS=Homo sapiens GN=YWHAG PE=1 SV=2	119	28285	7	6	25.5	247	0.95
58	14-3-3 protein epsilon OS=Homo sapiens GN=YWHAE PE=1 SV=1	118	29155	8	5	19.6	255	0.91
59	14-3-3 protein beta/alpha OS=Homo sapiens GN=YWHAB PE=1 SV=3	117	28065	6	4	17.5	246	0.75
60	Ras GTPase-activating-like protein IQGAP1 OS=Homo sapiens GN=IQGAP1 PE=1 SV=1	116	189134	8	7	4.3	1657	0.09
61	Phosphoglycerate mutase 1 OS=Homo sapiens GN=PGAM1 PE=1 SV=2	114	28786	8	6	28	254	1.15
62	Alkaline phosphatase, placental type OS=Homo sapiens GN=ALPP PE=1 SV=2	111	57917	7	6	16.4	535	0.25
63	Elongation factor 1-gamma OS=Homo sapiens GN=EEF1G PE=1 SV=3	111	50087	10	7	14.9	437	0.56
64	Gamma-enolase OS=Homo sapiens GN=ENO2 PE=1 SV=3	110	32930	6	5	21.4	285	0.47
65	L-lactate dehydrogenase A chain OS=Homo sapiens GN=LDHA PE=1 SV=2	108	36665	6	5	15.1	332	0.41
66	Hornerin OS=Homo sapiens GN=HRNR PE=1 SV=2	105	282228	4	3	2.1	2850	0.05
67	L-lactate dehydrogenase B chain OS=Homo sapiens GN=LDHB PE=1 SV=2	105	36615	5	4	14.1	334	0.41
68	Peroxiredoxin-2 OS=Homo sapiens GN=PRDX2 PE=1 SV=5	105	21878	7	4	21.2	198	0.77
69	Eukaryotic initiation factor 4A-I OS=Homo sapiens GN=EIF4A1 PE=1 SV=1	100	46125	10	9	22.9	406	0.51
70	Non-receptor tyrosine-protein kinase TYK2 OS=Homo sapiens GN=TYK2 PE=1 SV=3	99	133565	4	2	1.1	1187	0.05
71	Cofilin-1 OS=Homo sapiens GN=CFL1 PE=1 SV=3	98	18491	3	3	35.5	166	0.65
72	ATP-dependent RNA helicase DDX3X OS=Homo sapiens GN=DDX3X PE=1 SV=3	97	73198	4	4	8.3	662	0.19
73	Mitochondrial-processing peptidase subunit alpha OS=Homo sapiens GN=PMPCA PE=1 SV=2	95	58216	1	1	3.2	525	0.06
74	RNA-binding protein FUS OS=Homo sapiens GN=FUS PE=1 SV=1	95	53394	1	1	4.6	526	0.06
75	Heterogeneous nuclear ribonucleoprotein A1 OS=Homo sapiens GN=HNRNPA1 PE=1 SV=5	92	38723	7	5	15.6	372	0.39
76	Tubulin beta chain OS=Homo sapiens GN=TUBB PE=1 SV=2	92	49639	8	6	20.3	444	0.29
77	GTP-binding nuclear protein Ran OS=Homo sapiens GN=RAN PE=1 SV=3	90	24408	4	3	16.7	216	0.67
78	Keratin, type I cytoskeletal 14 OS=Homo sapiens GN=KRT14 PE=1 SV=4	88	51529	11	8	15.5	472	0.54
79	Tyrosine-protein kinase JAK1 OS=Homo sapiens GN=JAK1 PE=1 SV=2	88	133191	3	1	0.5	1154	0.02
80	14-3-3 protein theta OS=Homo sapiens GN=YWHAQ PE=1 SV=1	88	27747	3	3	13.1	245	0.41
81	Elongation factor 1-delta OS=Homo sapiens GN=EEF1D PE=1 SV=5	88	31103	3	3	15.3	281	0.23
82	Argininosuccinate synthase OS=Homo sapiens GN=ASS1 PE=1 SV=2	87	46501	9	5	9.7	412	0.41

83	ATP synthase subunit alpha, mitochondrial OS=Homo sapiens GN=ATP5A1 PE=1 SV=1	85	59714	5	3	4.9	553	0.17
84	T-complex protein 1 subunit theta OS=Homo sapiens GN=CCT8 PE=1 SV=4	84	59583	3	3	6.6	548	0.11
85	26S proteasome non-ATPase regulatory subunit 2 OS=Homo sapiens GN=PSMD2 PE=1 SV=3	83	100136	1	1	1.3	908	0.03
86	Fructose-bisphosphate aldolase C OS=Homo sapiens GN=ALDOC PE=1 SV=2	82	39431	4	3	9.1	364	0.08
87	Heat shock protein 75 kDa, mitochondrial OS=Homo sapiens GN=TRAP1 PE=1 SV=3	82	80060	2	1	2	704	0.08
88	Keratin, type I cytoskeletal 16 OS=Homo sapiens GN=KRT16 PE=1 SV=4	81	51236	9	6	12.3	473	0.37
89	Tubulin alpha-1C chain OS=Homo sapiens GN=TUBA1C PE=1 SV=1	80	49863	5	4	12.9	449	0.14
90	Fascin OS=Homo sapiens GN=FSCN1 PE=1 SV=3	80	54496	2	2	5.3	493	0.12
91	Heterogeneous nuclear ribonucleoprotein U OS=Homo sapiens GN=HNRNPU PE=1 SV=6	75	90528	2	2	4.5	825	0.07
92	Ezrin OS=Homo sapiens GN=EZR PE=1 SV=4	75	69370	7	6	8.7	586	0.2
93	Tubulin beta-4B chain OS=Homo sapiens GN=TUBB4B PE=1 SV=1	74	49799	6	6	20.2	445	0.29
94	Maestro heat-like repeat-containing protein family member 7 OS=Homo sapiens GN=MROH7 PE=2 SV=	73	145554	3	1	0.5	1323	0.02
95	GMP synthase [glutamine-hydrolyzing] OS=Homo sapiens GN=GMPS PE=1 SV=1	72	76667	3	3	7.9	693	0.09
96	Proteasome subunit alpha type-7 OS=Homo sapiens GN=PSMA7 PE=1 SV=1	72	27870	4	3	14.5	248	0.25
97	Epiplakin OS=Homo sapiens GN=EPPK1 PE=1 SV=2	71	555279	1	1	0.5	5090	0.01
98	Nucleosome assembly protein 1-like 1 OS=Homo sapiens GN=NAP1L1 PE=1 SV=1	71	45346	1	1	4.3	391	0.07
99	Keratin, type I cytoskeletal 19 OS=Homo sapiens GN=KRT19 PE=1 SV=4	71	44079	6	3	6.8	400	0.34
100	Glutathione S-transferase Mu 3 OS=Homo sapiens GN=GSTM3 PE=1 SV=3	71	26542	2	2	11.1	225	0.27
101	Serpin B6 OS=Homo sapiens GN=SERPINB6 PE=1 SV=3	69	42594	4	3	10.1	376	0.25
102	Keratin, type II cytoskeletal 6B OS=Homo sapiens GN=KRT6B PE=1 SV=5	69	60030	11	10	16.8	564	0.31
103	Annexin A4 OS=Homo sapiens GN=ANXA4 PE=1 SV=4	68	35860	3	3	11.6	319	0.3
104	Tubulin beta-2B chain OS=Homo sapiens GN=TUBB2B PE=1 SV=1	67	49921	7	5	18.2	445	0.21
105	Splicing factor, proline- and glutamine-rich OS=Homo sapiens GN=SFPQ PE=1 SV=2	66	76102	4	4	8.2	707	0.13
106	Stress-70 protein, mitochondrial OS=Homo sapiens GN=HSPA9 PE=1 SV=2	66	73635	4	4	7.7	679	0.14
107	ate dehydrogenase [ubiquinone] flavoprotein subunit, mitochondrial OS=Homo sapiens GN=SDHA PE=	66	72645	3	3	5.7	664	0.09
108	Neutral alpha-glucosidase AB OS=Homo sapiens GN=GANAB PE=1 SV=3	66	106807	5	5	7.2	944	0.13
109	Polyadenylate-binding protein 4 OS=Homo sapiens GN=PABPC4 PE=1 SV=1	65	70738	3	3	5.7	644	0.1
110	Carbamoyl-phosphate synthase [ammonia], mitochondrial OS=Homo sapiens GN=CPS1 PE=1 SV=2	65	164835	5	4	3.2	1500	0.08
111	Keratin, type II cytoskeletal 5 OS=Homo sapiens GN=KRT5 PE=1 SV=3	65	62340	10	9	15.3	590	0.29
112	Keratin, type I cytoskeletal 17 OS=Homo sapiens GN=KRT17 PE=1 SV=2	65	48076	9	6	14.1	432	0.49
113	Non-POU domain-containing octamer-binding protein OS=Homo sapiens GN=NONO PE=1 SV=4	64	54197	3	3	8.3	471	0.19
114	Far upstream element-binding protein 1 OS=Homo sapiens GN=FUBP1 PE=1 SV=3	63	67518	2	2	2.6	644	0.1
115	Electron transfer flavoprotein subunit alpha, mitochondrial OS=Homo sapiens GN=ETFA PE=1 SV=1	63	35058	3	1	5.7	333	0.09
116	APC membrane recruitment protein 1 OS=Homo sapiens GN=AMER1 PE=1 SV=2	62	123952	19	1	0.9	1135	0.03
117	SUMO-activating enzyme subunit 1 OS=Homo sapiens GN=SAE1 PE=1 SV=1	62	38426	1	1	3.8	346	0.09
118	Glucosidase 2 subunit beta OS=Homo sapiens GN=PRKCSH PE=1 SV=2	62	59388	1	1	2.1	528	0.06
119	Leucine-rich PPR motif-containing protein, mitochondrial OS=Homo sapiens GN=LRPPRC PE=1 SV=3	62	157805	5	3	2.1	1394	0.06
120	3-hydroxyacyl-CoA dehydrogenase type-2 OS=Homo sapiens GN=HSD17B10 PE=1 SV=3	62	26906	4	4	18.4	261	0.6
121	Peptidyl-prolyl cis-trans isomerase A OS=Homo sapiens GN=PPIA PE=1 SV=2	62	18001	2	2	7.9	165	0.19
122	26S protease regulatory subunit 8 OS=Homo sapiens GN=PSMC5 PE=1 SV=1	62	45597	1	1	3.2	406	0.07
123	Delta-1-pyrroline-5-carboxylate synthase OS=Homo sapiens GN=ALDH18A1 PE=1 SV=2	61	87248	2	1	1.5	795	0.04
124	Polyadenylate-binding protein 1 OS=Homo sapiens GN=PABPC1 PE=1 SV=2	61	70626	2	2	4.9	636	0.1
125	Radixin OS=Homo sapiens GN=RDX PE=1 SV=1	61	68521	7	6	9.4	583	0.26
126	Peroxiredoxin-6 OS=Homo sapiens GN=PRDX6 PE=1 SV=3	60	25019	3	3	17.4	224	0.46
127	ATP-dependent RNA helicase DDX39A OS=Homo sapiens GN=DDX39A PE=1 SV=2	59	49098	2	2	2.6	427	0.07
128	Filamin-A OS=Homo sapiens GN=FLNA PE=1 SV=4	59	280564	3	3	1.9	2647	0.02
129	Fumarate hydratase, mitochondrial OS=Homo sapiens GN=FH PE=1 SV=3	58	54602	5	4	13.1	510	0.19
130	S-adenosylmethionine synthase isoform type-2 OS=Homo sapiens GN=MAT2A PE=1 SV=1	57	43633	3	3	9.4	395	0.24
131	4F2 cell-surface antigen heavy chain OS=Homo sapiens GN=SLC3A2 PE=1 SV=3	57	67952	5	4	7.6	630	0.21
132	Transportin-1 OS=Homo sapiens GN=TNPO1 PE=1 SV=2	57	102289	3	2	2.6	898	0.06
133	Plastin-3 OS=Homo sapiens GN=PLS3 PE=1 SV=4	57	70766	2	2	4	630	0.05
134	Coatomer subunit delta OS=Homo sapiens GN=ARCN1 PE=1 SV=1	57	57174	1	1	2.2	511	0.06
135	Poly(rC)-binding protein 2 OS=Homo sapiens GN=PCBP2 PE=1 SV=1	56	38556	2	2	5.2	365	0.18
136	Transaldolase OS=Homo sapiens GN=TALDO1 PE=1 SV=2	56	37516	5	4	12.2	337	0.29
137	Chloride intracellular channel protein 1 OS=Homo sapiens GN=CLIC1 PE=1 SV=4	56	26906	3	3	16.2	241	0.42
138	Rootletin OS=Homo sapiens GN=CROCC PE=1 SV=1	56	228388	2	2	0.7	2017	0.01
139	Thioredoxin reductase 1, cytoplasmic OS=Homo sapiens GN=TXNRD1 PE=1 SV=3	55	70862	3	3	6.6	649	0.15
140	Eukaryotic translation initiation factor 3 subunit I OS=Homo sapiens GN=EIF3I PE=1 SV=1	55	36479	2	2	6.5	325	0.19
141	Ubiquilin-2 OS=Homo sapiens GN=UBQLN2 PE=1 SV=2	55	65655	2	2	3.7	624	0.1
142	Pyruvate carboxylase, mitochondrial OS=Homo sapiens GN=PC PE=1 SV=2	54	129551	4	3	2.2	1178	0.03
143	Keratin, type II cytoskeletal 6C OS=Homo sapiens GN=KRT6C PE=1 SV=3	54	59988	11	10	16.8	564	0.24
144	Keratin, type I cytoskeletal 27 OS=Homo sapiens GN=KRT27 PE=1 SV=2	54	49792	3	2	3.9	459	0.14
145	Staphylococcal nuclease domain-containing protein 1 OS=Homo sapiens GN=SND1 PE=1 SV=1	54	101934	5	5	3.8	910	0.13
146	Transitional endoplasmic reticulum ATPase OS=Homo sapiens GN=VCP PE=1 SV=4	54	89266	4	4	7.1	806	0.16
147	Dermcidin OS=Homo sapiens GN=DCD PE=1 SV=2	54	11277	3	1	12.7	110	0.3
148	Inosine-5~-monophosphate dehydrogenase 2 OS=Homo sapiens GN=IMPDH2 PE=1 SV=2	53	55770	3	3	4.9	514	0.19
149	Proteasome subunit alpha type-5 OS=Homo sapiens GN=PSMA5 PE=1 SV=3	53	26394	2	2	12	241	0.13
150	Voltage-dependent anion-selective channel protein 1 OS=Homo sapiens GN=VDAC1 PE=1 SV=2	53	30754	1	1	3.9	283	0.11
151	Annexin A3 OS=Homo sapiens GN=ANXA3 PE=1 SV=3	53	36353	5	5	18.3	323	0.3
