# Supplementary material for: Long noncoding RNA AGPG regulates PFKFB3-mediated tumor glycolytic reprogramming
Source: Nat Commun. 2020 Mar 20;11:1507. doi: 10.1038/s41467-020-15112-3 (PMC7083971; doi:10.1038/s41467-020-15112-3)
Supplement: Supplementary file 6 — Reporting Summary [file 41467_2020_15112_MOESM6_ESM.pdf]

nature research

Corresponding author(s): Huai-Qiang Ju

Last updated by author(s): Feb 2, 2020

Reporting Summary

Nature Research wishes to improve the reproducibility of the work that we publish. This form provides structure for consistency and transparency in reporting. For further information on Nature Research policies, see Authors & Referees and the Editorial Policy Checklist.

Statistics

For all statistical analyses, confirm that the following items are present in the figure legend, table legend, main text, or Methods section.

n/a	Confirmed	
☐	☒	The exact sample size (*n*) for each experimental group/condition, given as a discrete number and unit of measurement
☐	☒	A statement on whether measurements were taken from distinct samples or whether the same sample was measured repeatedly
☐	☒	The statistical test(s) used AND whether they are one- or two-sided<br>*Only common tests should be described solely by name; describe more complex techniques in the Methods section.*
☒	☐	A description of all covariates tested
☐	☒	A description of any assumptions or corrections, such as tests of normality and adjustment for multiple comparisons
☐	☒	A full description of the statistical parameters including central tendency (e.g. means) or other basic estimates (e.g. regression coefficient) AND variation (e.g. standard deviation) or associated estimates of uncertainty (e.g. confidence intervals)
☐	☒	For null hypothesis testing, the test statistic (e.g. *F*, *t*, *r*) with confidence intervals, effect sizes, degrees of freedom and *P* value noted<br>*Give P values as exact values whenever suitable.*
☒	☐	For Bayesian analysis, information on the choice of priors and Markov chain Monte Carlo settings
☒	☐	For hierarchical and complex designs, identification of the appropriate level for tests and full reporting of outcomes
☐	☒	Estimates of effect sizes (e.g. Cohen's *d*, Pearson's *r*), indicating how they were calculated

Our web collection on statistics for biologists contains articles on many of the points above.

Software and code

Policy information about availability of computer code

Data collection	The posttranslational modification data of PFKFB3 were obtained from the dbPAF (version 1.0) and CPLM databases (version 1.0).
Data analysis	The experimental results were analyzed using GraphPad Prism 8.0.1. DESeq2 (version 3.10) was employed for differential expression analysis. The pathway analysis was performed with GSEA (version 4.0.3), while JASPAR software (version 2018) predicted the p53 binding sequence. The posttranslational modification data of PFKFB3 were obtained from the dbPAF (version 1.0) and CPLM databases (version 1.0) and visualized by IBS software (version 1.0.3). For CLIP-seq analysis, HomeR (homer2 version) was used to perform the motif analysis on the binding peaks obtained by the Piranha (version 1.2.1) and CIMS (version 1.1.3) analyses.

For manuscripts utilizing custom algorithms or software that are central to the research but not yet described in published literature, software must be made available to editors/reviewers. We strongly encourage code deposition in a community repository (e.g. GitHub). See the Nature Research guidelines for submitting code & software for further information.

Data

Policy information about availability of data

All manuscripts must include a data availability statement. This statement should provide the following information, where applicable:

- Accession codes, unique identifiers, or web links for publicly available datasets
- A list of figures that have associated raw data
- A description of any restrictions on data availability

Expression and survival analyses for lncRNAs in ESCC were performed using data obtained from TCGA55 (http://www.cbioportal.org/publicportal/). DESeq2 (version 3.10) was employed for differential expression analysis. The pathway analysis was performed with GSEA (version 4.0.3), while JASPAR software (version 2018) predicted the p53 binding sequence. The posttranslational modification data of PFKFB3 were obtained from the dbPAF (version 1.0) and CPLM databases (version 1.0) and visualized by IBS software (version 1.0.3). The CLIP-Seq dataset is availible at NCBI Sequence Read Archive (SRA) under BioProject PRJNA591321 (https://www.ncbi.nlm.nih.gov/bioproject/591321). For CLIP-seq analysis, HomeR (homer2 version) was used to perform the motif analysis on the binding peaks obtained by

the Piranha (version 1.2.1) and CIMS (version 1.1.3) analyses. All the other data supporting the findings of this study are available within the article and its Supplementary Information Files or from the corresponding authors upon reasonable request.

Field-specific reporting

Please select the one below that is the best fit for your research. If you are not sure, read the appropriate sections before making your selection.

☒ Life sciences ☐ Behavioural & social sciences ☐ Ecological, evolutionary & environmental sciences

For a reference copy of the document with all sections, see nature.com/documents/nr-reporting-summary-flat.pdf

Life sciences study design

All studies must disclose on these points even when the disclosure is negative.

Sample size	No statistical methods were used to predetermine sample size. Sample size are based on a lot of previous publications and our previous experience, which is the most optimal to generate statistically significant results. All experiments were carried out at least three times. For each experiment, n=3 biologically independent samples unless otherwise stated.
Data exclusions	No data were excluded from the analyses.
Replication	All experiments were carried out at least three times. For each experiment, n=3 biologically independent samples unless otherwise stated.
Randomization	In general, the samples/cells were randomized into different groups prior to treatment. For animal study, all the animals were randomly grouped for experiments.
Blinding	The investigators were not blinded to group allocation, because the experimental design was complicated, the researchers were limited, and blinding feasibility was poor.

Reporting for specific materials, systems and methods

We require information from authors about some types of materials, experimental systems and methods used in many studies. Here, indicate whether each material, system or method listed is relevant to your study. If you are not sure if a list item applies to your research, read the appropriate section before selecting a response.

Materials & experimental systems

n/a	Involved in the study	
☐	☒	Antibodies
☐	☒	Eukaryotic cell lines
☒	☐	Palaeontology
☐	☒	Animals and other organisms
☐	☒	Human research participants
☒	☐	Clinical data

Methods

n/a	Involved in the study	
☒	☐	ChIP-seq
☐	☒	Flow cytometry
☒	☐	MRI-based neuroimaging

Antibodies

Antibodies used	Anti-GAPDH (Cell Signaling Technology, Beverly, USA, 5174), anti-vinculin (Cell Signaling Technology, 13901), anti-PFKFB3 (Abcam, Cambridge, MA, USA, ab181861), anti-PFKFB3 (phospho S461)(ab232498), anti-p27 (Abcam, ab32034), anti-CDK1 (Abcam, ab133327), anti-p53 (Abcam, ab1101), anti-p21 (Abcam, ab109199), anti-CDK3 (Abcam, ab96847), anti-CDK6 (Abcam, ab124821), anti-FLAG tag (Cell Signaling Technology, 8146), anti-His tag (Abcam, ab9108), anti-Cdc27 (Abcam, ab10538), and anti-ubiquitin antibodies (Cell Signaling Technology, 3933) were used in this study.
Validation	Based on the information on the manufacturers' website, antibody was validated in wild-type and knockout samples using WB experiments, and was supported by multiple publications. Additional validation was performed by ourselves using siRNA-treated samples and control samples.

Eukaryotic cell lines

Policy information about cell lines

Cell line source(s)	Het-1A and HCT-116 cells were obtained from American Type Culture Collection (ATCC, Rockville, MD, USA). KYSE30, KYSE510, KYSE180, KYSE150, KYSE520, KYSE70 and KYSE180 cells were obtained from German Cell Culture Collection (DSMZ, Braunschweig, Germany). TE-1, TE-9 and TE-15 cells were obtained from the Cell Bank of Shanghai Institute of Cell Biology

	(Chinese Academy of Medical Sciences, Shanghai, China). HCT-116 (p53 KO) and 293T inducible K-RasG12V (iK-RasG12V) cells were provided by Prof. Peng Huang (SYSUCC, Guangzhou, China).
Authentication	Based on short tandem repeat (STR) profiling supplied by vendors, no cells used in this study are found in the database of commonly misidentified cell lines. All cells, including HCT-116 (p53 KO) and 293T inducible K-RasG12V, were further authenticated via STR-PCR DNA profiling by Guangzhou Cellcook Biotech Co.,Ltd. (Guangzhou, China),
Mycoplasma contamination	All cells were determined to be free of mycoplasma contamination.
Commonly misidentified lines (See ICLAC register)	No cells used in this study are found in the database of commonly misidentified cell lines.

Animals and other organisms

Policy information about studies involving animals; ARRIVE guidelines recommended for reporting animal research

Laboratory animals	4-week-old female BALB/c nu/nu mice (5 mice per group)
Wild animals	The study did not involve wild animals
Field-collected samples	The study did not involve Field-collected samples
Ethics oversight	The animal studies were approved by the Institutional Animal Care and Use Committee of Sun Yat-Sen University.

Note that full information on the approval of the study protocol must also be provided in the manuscript.

Human research participants

Policy information about studies involving human research participants

Population characteristics	All clinicopathological information including Age, Gender, Differentiation status, Tumor depth, Lymph node invasion, Vascular invasion, Distant metastasis and Clinical stage is provided in Supplementary Table 1,4,5.
Recruitment	Clinical samples were collected from SYSUCC (Guangzhou, China). All patients had a histological diagnosis of cancer. After the operation, the patients received regular follow-up. There may be selection bias, but this bias has little effect on the results of this study. Because the results (AGPG expression or survival analysis) have been further confirmed using data obtained from TCGA and samples from multiple cancer types.
Ethics oversight	The study was approved by the Medical Ethics Committee of SYSUCC. Written informed consent was obtained from each patient who provided samples.

Note that full information on the approval of the study protocol must also be provided in the manuscript.

Flow Cytometry

Plots

Confirm that:

☒ The axis labels state the marker and fluorochrome used (e.g. CD4-FITC).

☒ The axis scales are clearly visible. Include numbers along axes only for bottom left plot of group (a 'group' is an analysis of identical markers).

☒ All plots are contour plots with outliers or pseudocolor plots.

☒ A numerical value for number of cells or percentage (with statistics) is provided.

Methodology

Sample preparation	Cells were trypsinized and re-suspended in 200 μl cold PBS, 5 ml of cold 90% ethanol was added for fixation overnight. Prior to the assay, cells were centrifuged for 5 min at 200 × g and re-suspended in 0.5 ml PBS with propidium iodide and RNase A, incubating 30 min at 37 °C.
Instrument	Gallio flow cytometer (Beckman Coulter, CA, USA)
Software	Flowjo7.6.5
Cell population abundance	Approximately 1 million per sample
Gating strategy	The gating strategy is used to exclude cell debris and aggregates. The gating strategy is provided in the Supplementary Table 7.

☒ Tick this box to confirm that a figure exemplifying the gating strategy is provided in the Supplementary Information.
